# Functional interactions in patients with hemianopia: A graph theory-based connectivity study of resting fMRI signal

**DOI:** 10.1371/journal.pone.0226816

**Published:** 2020-01-06

**Authors:** Caterina A. Pedersini, Joan Guàrdia-Olmos, Marc Montalà-Flaquer, Nicolò Cardobi, Javier Sanchez-Lopez, Giorgia Parisi, Silvia Savazzi, Carlo A. Marzi

**Affiliations:** 1 Physiology and Psychology Section, Department of Neuroscience, Biomedicine and Movement Sciences, University of Verona, Verona, Italy; 2 Department of Social Psychology and Quantitative Psychology, School of Psychology, Institute of Neuroscience, Institute of Complex Systems, University of Barcelona, Barcelona, Spain; 3 Department of Social Psychology and Quantitative Psychology, School of Psychology, Institute of Complex Systems, University of Barcelona, Barcelona, Spain; 4 Perception and Awareness (PandA) Laboratory, Department of Neuroscience, Biomedicine and Movement Sciences, University of Verona, Verona, Italy; 5 National Institute of Neuroscience, Verona, Italy; University of Maryland at College Park, UNITED STATES

## Abstract

The assessment of task-independent functional connectivity (FC) after a lesion causing hemianopia remains an uncovered topic and represents a crucial point to better understand the neural basis of blindsight (i.e. unconscious visually triggered behavior) and visual awareness. In this light, we evaluated functional connectivity (FC) in 10 hemianopic patients and 10 healthy controls in a resting state paradigm. The main aim of this study is twofold: first of all we focused on the description and assessment of density and intensity of functional connectivity and network topology with and without a lesion affecting the visual pathway, and then we extracted and statistically compared network metrics, focusing on functional segregation, integration and specialization. Moreover, a study of 3-cycle triangles with prominent connectivity was conducted to analyze functional segregation calculated as the area of each triangle created connecting three neighboring nodes. To achieve these purposes we applied a graph theory-based approach, starting from Pearson correlation coefficients extracted from pairs of regions of interest. In these analyses we focused on the FC extracted by the whole brain as well as by four resting state networks: The Visual (VN), Salience (SN), Attention (AN) and Default Mode Network (DMN), to assess brain functional reorganization following the injury. The results showed a general decrease in density and intensity of functional connections, that leads to a less compact structure characterized by decrease in functional integration, segregation and in the number of interconnected *hubs* in both the Visual Network and the whole brain, despite an increase in long-range inter-modules connections (occipito-frontal connections). Indeed, the VN was the most affected network, characterized by a decrease in intra- and inter-network connections and by a less compact topology, with less interconnected nodes. Surprisingly, we observed a higher functional integration in the DMN and in the AN regardless of the lesion extent, that may indicate a functional reorganization of the brain following the injury, trying to compensate for the general reduced connectivity. Finally we observed an increase in functional specialization (lower between-network connectivity) and in inter-networks functional segregation, which is reflected in a less compact network topology, highly organized in functional clusters. These descriptive findings provide new insight on the spontaneous brain activity in hemianopic patients by showing an alteration in the intrinsic architecture of a large-scale brain system that goes beyond the impairment of a single RSN.

## Introduction

Hemianopia is a visual field defect resulting in loss of vision in half of the visual field of both eyes, as consequence of a contralateral post-chiasmatic lesion of the visual pathways and occipital cortex. Depending on the specific lesion site and extent, the visual defect could affect one entire hemifield (lateral homonymous hemianopia) or a single visual quadrant (quadrantanopia) [1]. Functional recovery usually occurs within 2–3 months and is unlikely after 6 months following brain injury [2]. Importantly, some patients retain residual visual sensitivity without conscious perception in their otherwise blind hemifield. This phenomenon has been termed “blindsight” that is, the ability to detect, localize or discriminate visual stimuli without being aware of them [3]. Over the past 30 years, task-related brain activity [4] and electrophysiological signals [5] have been recorded during visual stimulus presentation to the blind hemifield of these patients with the aim of exploring the neural mechanisms of unconscious vision. In these experiments, patients are asked to give a response even if they cannot consciously perceive anything in the blind visual field. Moving stimuli have often been used to study blindsight as visual motion has been shown to be a perceptual feature that can increase the probability of finding this phenomenon.

Diffusion Tensor Imaging (DTI) has been widely employed to assess structural connectivity [6,7] by evaluating the integrity of white matter fibers interconnecting cortical and subcortical structures. This technique helps in assessing the neural bases of blindsight and enables the rapid and non-invasive construction of structural brain connectivity maps. Partial integrity of white matter fibers connecting subcortical structures as the Lateral Geniculate Nucleus (LGN) with ipsi- and contra-lateral extrastriate visual areas as the human Motion Complex (area hMT) have been observed in hemianopic patients with but not in those without blindsight. Therefore, this subcortico-cortical white matter fiber can represent a possible anatomical prerequisite of this phenomenon.

An important aspect that has to be considered when studying brain damaged patients is represented by functional recovery, that represents the behavioural compensation following brain injury, mediated by brain plasticity [8]. Neuroplasticity is the ability of the nervous system to change its structural and functional organization during the whole life and to react by optimizing the neural network in response to a brain injury [9]. As an example, motor recovery after a stroke represents a plastic phenomenon by which focal injuries determine intra- or inter-hemispheric alterations and changes in the connections between brain nodes within the motor network [10,11]. Reorganization of the visual system following brain damage has been widely demonstrated. Different mechanisms can mediate neuroplasticity. They can be represented by changes in the ipsilesional primary visual cortex in terms of disinhibition or creation of new long-range connections [12,13], in the contralesional hemisphere [14], in some visual areas in the ipsilesional hemisphere [15] or in the functional connections among extrastriate areas and V1 [16]. (For a review see [17,18]).

New rehabilitation techniques aim to take advantage of neuroplasticity to help patients recovering some impaired cognitive skills (e.g. Visual Restoration Therapy for hemianopic patients).[18] Therefore, the assessment of brain plasticity following a stroke by means of a connectivity-based approach could help in understanding the functional reorganization of the brain, providing new insights into brain strategies for recovery based on the conception of the brain as a complex system [19].

Functional connectivity represents the undirected statistical dependency between time courses extracted from different brain regions during the execution of a task or during resting state [20]. Neuroimaging techniques allow to measure functional connectivity by assessing the level of co-activation and similarity of spontaneous low-frequency oscillations [21,22]. These techniques have widely demonstrated that the temporal correlation between regions of interest reproduces the ongoing functional interconnectivity [23]. Resting state (RS) fMRI focuses on synchronous, spontaneous, low frequency fluctuations (<0.1 Hz) in the BOLD signal, occurring when participants are instructed to relax without engaging in a specific task.

Few studies have been carried out on the assessment of brain functional reorganization in hemianopic patients, most of them using multichannel electroencephalography [24,25]. In contrast, the impact of different diseases on functional connectivity has been widely studied in patients with neurodegenerative disorders, as Alzheimer [26], psychiatric disorders such as schizophrenia [27,28], depression [29,30] and obsessive compulsive disorder [31]; and, neurodevelopmental disorders such as autism [32] and attention deficit hyperactivity [33]. In these studies, RS functional connectivity represents an ideal tool to assess the remote physiological effects of a lesion on distant but functionally connected areas of the brain (“*distributed injury hypothesis*”) by evaluating alterations in their synchronization as a consequence of the disease [19]. These changes in brain synchronization caused by the lesion can constrain specific behavioral outputs, influencing the way in which regions are recruited and communicate during behavior [34].

Group RS studies have shown the existence of reliable and stable functionally linked networks during rest, referred as resting-state networks or RSN [35–37] mostly “named” on the basis of the spatial similarity between resting state networks and activation patterns during task-dependent fMRI experiments. The most fundamental and clearly identified RSN in the literature is represented by the Default Mode Network (DMN), which activates during rest and deactivates during the execution of a task [38]. It is mainly linked to core processes of human cognition such as integration of cognitive and emotional processes and mind-wandering. The main areas of the DMN are the medial posterior frontal cortex, the posterior cingulate cortex, the inferior parietal lobe and the hippocampus. DMN alterations have been reported in many neurological and neuropsychiatric disorders such as Alzheimer disease [39] and Schizophrenia [40]. Other resting state networks in the brain are the salience, auditory, basal ganglia, higher and primary visual, visuospatial, language, executive and sensorimotor network [41].

The assessment of neuroplasticity as well as of task-independent functional connectivity after a stroke causing hemianopia remains an uncovered topic to be explored. Therefore, the main aim of our study is twofold: first of all we explored and described in detail the RS functional connectivity in an heterogeneous group of patients who share the same diagnosis of permanent hemianopia, and then we assessed the difference with a group of age-matched healthy participants in the synchronization of brain activation during RS through the extraction of graph summary measures.

According to the specific lesion location and patients’ symptoms, we focused on the evaluation of intra- and inter-networks functional connectivity considering four main RSNs: the Visual Network (VN), directly damaged by the lesion and that determines the phenotype of our clinical population; the Salience (SN) and the Attentional network (AN) [42], as the injury of some of our patients involves fronto-parieto-temporal areas, despite the absence of symptoms directly related to their damage, and the Default Mode Network (DMN) [43], a control network, not related to the specific phenotype of the experimental group.

Among the different methods that can be applied to study RS functional connectivity, we decided to use a graph theory-based connectivity approach as it provides a way to assess the network topology by reducing the brain network to a simplified set of nodes and edges [44–47]. It enables the extraction and comparison of summary measures of brain topology and efficiency in healthy and in clinical populations [48]. For all these reasons, this approach has been widely used to assess the intrinsic network organization in psychiatric and neurological syndromes [37,49,50]. Furthermore, it allows to assess between-groups differences in functional segregation, that is the organization of functionally related brain regions in modules with high within-modules and low between-modules density, and functional integration, that is the strong coupling between modules. Balanced levels of functional segregation and integration have demonstrated to be necessary to maintain high cognitive functions. [51–53].

For each group we extracted five summary measures of functional integration and segregation, that allow to assess both small-world properties and network efficiency. Indeed, clustering coefficient and characteristic path length are small-world parameters [54], whilst local and global efficiency are parameters of network efficiency [55] that allow to assess functional segregation and integration, respectively. In addition, we extracted node degree as a measure of node connectedness in the network [56].

In summary, the specific purpose of the present study is to shed light on alterations in brain plasticity and in intrinsic cerebral functional architecture following different kinds of lesion affecting the visual system and causing a similar visual impairment. In this respect, this study represents a preliminary step toward the assessment of a general pattern of functional connectivity in a specific clinical population.

## Material and methods

### Subjects

Ten hemianopic patients (3 females; mean age = 54.5 years, SD = 13.14; see Table 1), right-handed, with long-standing post-chiasmatic lesions (median post-lesion time = 32.7 months) causing visual field loss, and a group of 10 healthy participants (6 females; mean age = 54.9, SD = 13.61) with no history of neurological disorders were recruited. No significant difference was found between the two groups considering the years of schooling (Wilcoxon Test: μ patients = 12, SD = 2.71; μ controls = 13.9, SD = 2.35; Z = -1.39; p = 0.1642). Patients were recruited from the hospitals of Verona and Treviso. The visual field was examined with the Humprey field analyser that assesses the monocular visual field using a flash detection task (HFA 750i, Zeiss-Humprey, Leandro, CA, USA). The exclusion criteria included past or present neurologic disorders other than those related to hemianopia, psychiatric disorders, drugs or alcohol addiction, general cognitive impairment, and deficit of spatial attention (i.e. hemineglect). All participants were right handed and had normal or corrected-to-normal visual acuity. A brief description of patients’ lesion location, time and type of event can be found in Table 1. Written informed consent was obtained after participants had been fully informed about the testing procedures and their rights. The study was approved by the Ethics Committee of the European Research Council and of the Azienda Ospedaliera Universitaria Integrata of Verona (AOUI).

**Table 1:** Patient’s clinical description.

Patient (birth date/gender)	Lesion/Visual Deficit	Time elapsed post injury– T1-weighted image	Time elapsed post injury–Resting State acquisition
**PT01** **(1967/F)**	*Neuroradiological Description*: Lesion involving the median para-sagittal portion of the left occipital lobe. The lesion involves the lingual gyrus, with peri-calcarine fissure distribution. *Visual Defect*: Right lateral homonymous hemianopia. Ischemic Stroke: 04/2009 T1-weighted image: 02/2017	92 months	100 months
**PT02** **(1966/F)**	*Neuroradiological Description*: Lesion involving the right temporal, parietal and occipital lobe. In the occipital lobe, the lesion involves the superior and a portion of the middle occipital gyri with interruption of the right optic radiation. *Visual Defect*: Left lateral homonymous hemianopia. Hemorrhagic head injury: 06/2014 T1-weighted image: 03/2017	33 months	38 months
**PT03** **(1965/F)**	*Neuroradiological Description*: Ischemic lesion involving the grey matter of the anterior half of right calcarine fissure up to the origin of parieto-occipital fissure. *Visual Defect*: Upper left homonymous quadrantanopia. Ischemic Stroke: 07/2012 T1-weighted image: 04/2017	57 months	62 months
**PT04** **(1948/M)**	*Neuroradiological Description*: Lesion involving the medial portion of right occipital lobe, with an extension over the parieto-occipital fissure. An important involvement of the lingual and fusiform gyri till the occipital pole, with alterations of the calcarine fissure is observed. *Visual Defect*: Lower left homonymous quadrantanopia. Ischemic Stroke: 08/2016 T1-weighted image:05/2017	9 months	13 months
**PT05** **(1961/M)**	*Neuroradiological Description*: Lesion involving the left infero-lateral part of the occipital lobe with extension in the lingual and fusiform giri. Laterally, the lesions is below the lateral occipital sulcus. *Visual Defect*: Upper right homonymous quadrantanopia. Hemorrhagic head injury: 06/2016 T1-weighted image: 10/2017	16 months	17 months
**PT06** **(1996/M)**	*Neuroradiological Description*: Large ischemic lesion in all vascular territory of right middle cerebral artery, involving frontal, temporal and parietal lobes on this side. *Visual Defect*: Left lateral homonymous hemianopia with a greater visual deficit in the lower quadrant. Hemorrhagic head injury: 04/2014 T1-weighted image: 09/2017	41 months	41 months
**PT07** **(1952/M)**	*Neuroradiological Description*: Ischemic lesion of part of the vascular territory of right posterior cerebral artery. The alteration involves the calcarine fissure, the lingual and the fusiform giri. *Visual Defect*: Left lateral homonymous hemianopia Ischemic Stroke: 10/2017 T1weighted image: 05/2018	7 months	7 months
**PT08** **(1961/M)**	*Neuroradiological Description*: Lesion involving the left temporo-parietal lobe, with extension to the occipital lobe in the superior and middle occipital gyri. The alteration of the white matter in the occipital lobe suggests an involvement of the upper part of left optic radiation. *Visual Defect*: Right lateral homonymous hemianopia. Hemorrhagic head injury: 11/2013 T1-weighted image: 04/2017	41 months	47 months
**PT09** **(1965/M)**	*Neuroradiological Description*: Ischemic lesion of part of the vascular territory of the left posterior cerebral artery. The alteration involves the anterior and middle portion of calcarine fissure, the lingual gyrus and the posterior part of fusiform gyrus. *Visual Defect*: Right lateral homonymous hemianopia. Ischemic Stroke: 02/2017 T1-weighted image: 07/2017	5 months	17 months
**PT10** **(1954/F)**	*Neuroradiological Description*: Ischemic lesion in the vascular portion of the left posterior cerebral artery, involving the entire occipital lobe, including the left calcarine fissure. *Visual Defect*: Lower right homonymous quadrantanopia. Ischemic Stroke: 05/2016 T1-weighted image: 07/ 2018	26 months	26 months

Neuroradiological description of the lesion, type and onset of the injury, date of the acquisition of the T1-weighted image used for the neuroradiological description, time elapsed from the injury to the structural scanning session and from the injury to the resting state data acquisition.

#### Neuropsychological assessment

Cognitive functioning in relation to age and education, and impairments of spatial attention were evaluated through a neuropsychological battery of tests, including the Mini-Mental State Examination (MMSE) [57] the Line Bisection Test [58] the Diller letter H cancellation Test and the Bell Cancellation Test [59,60]. The Unilateral Neglect Syndrome is a neuropsychological condition characterized by the impairment in shifting the attention toward the contralateral side of the body or of the environment without visual loss. It could be merged or confused with Lateral Homonymous Hemianopia as some clinical symptoms appear similar. For this reason, it is important to differentially diagnose these two neuropsychological impairments. Therefore, we carried out an assessment of the presence of unilateral spatial neglect by using: Line Bisection test taken from the Behavioural Inattention Test [58] where a score > 7 indicates the presence of Neglect, the Diller letter H cancellation [59] and the Bell Cancellation Test [60] where a score of 5 unmarked elements indicates the presence of Neglect. The MMSE is a 30-item screening tool designed to quickly evaluate the integrity of normal cognitive functioning according to age and years of education [57]. The maximum score obtained is 30 and 24 is the established cut-off point to determine the presence of deterioration [61]. These tests were performed with the general aim of excluding patients with cognitive decline and neuropsychological impairments other than hemianopia. For this reason, no statistical analysis was performed on these data. No patient showed cognitive decline (median = 29.24, std = 1.79) or other neuropsychological impairments apart from hemianopia. Finally, patient’s subjective impressions on their visual abilities in everyday life were assessed with the Visual Function Questionnaire (VFQ25) [62].

#### Lesion details

**Fig 1:**
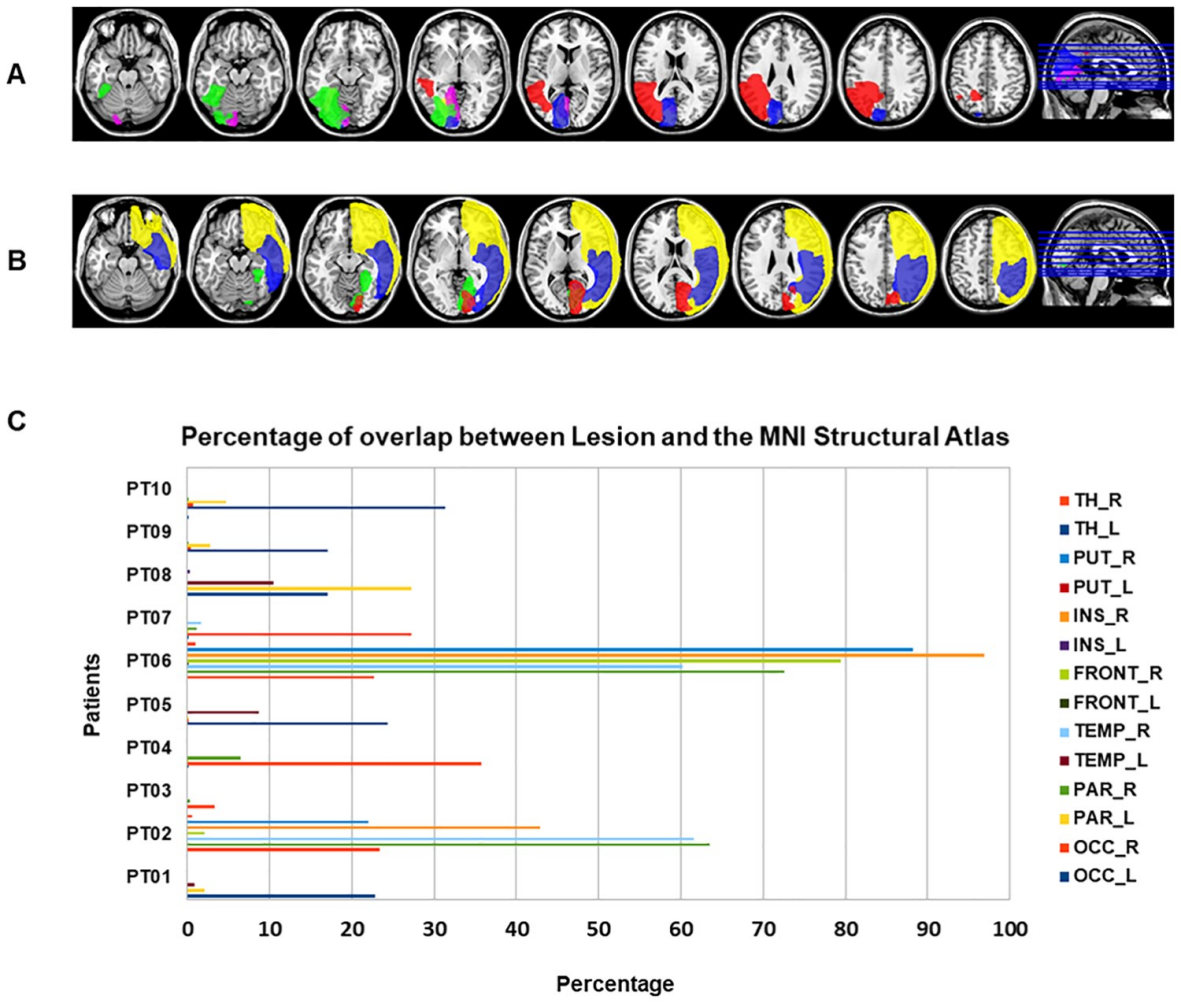
Representation of each patients’ lesion. Overlapped lesions of left (A) and right (B) damaged patients on the ch2.nii template of MRICron, represented on multislices. Each color represents one patient. (C). Quantification of the percentage of overlap between each patient’s lesion and the MNI Structural Atlas of fsl. TH: Thalamus; PUT = Putamen; INS = Insula; FRONT = Frontal Lobe; TEMP = Temporal Lobe; PAR = Parietal Lobe; OCC = Occipital Lobe.

Considering the heterogeneity of our sample and the availability of a 1mm^3^ isotropic T1-weighted image acquired for each patient, we created a mask of each lesion to better visualize and quantify its location and extent. Thus, starting from the bias-field corrected T1-weighted image, we created a mask of each lesion using the software ITK-SNAP [63]. Once created, the lesion mask was normalized to the standard MNI space with a spatial resolution of 1mm, using linear transformation (FLIRT). The software MRICron [64] was used to create two images representing the overlap between left or right brain lesions on the ch2.nii template brain [65] (see Fig 1). Lesion volumes, total intracranial volume and the percentage of damaged voxels are shown in Table 2.

**Table 2:** Patient lesion details.

PATIENT	Total Lesion Volume (mm^3^)	Intracranial Volume	Brain Damage (%)
PT01	27770	1958323	1.42
PT02	254848	1959137	13.01
PT03	3108	1998194	0.16
PT04	39912	1942996	2.05
PT05	38217	1955847	1.95
PT06	752786	1955749	38.49
PT07	26573	2013290	1.32
PT08	85802	2008821	4.27
PT09	22067	1919340	1.15
PT10	39344	1980551	1.99

Lesion volumes, total intracranial volume and the percentage of damaged voxels.

Thus, we extracted lateralized regions of interest from the MNI structural atlas of fsl [66,67] corresponding to the occipital, temporal, parietal and frontal lobe and to the insula, putamen and thalamus. These ROIs were thresholded to avoid the overlap between regions that could overestimate the real size of the lesion. The overlap between these masks and the lesion was quantified as the percentage of voxels (see Fig 1) and a visual inspection was performed based on the AAL Atlas to better describe which regions were directly affected by the lesion.

As can be seen from Fig 1, all patients except PT02, PT06 and PT08 have a lesion mainly involving the occipital lobe and, more in detail, the calcarine cortex, the cuneus, the lingual gyrus and the fusiform gyrus. In addition to that in occipital lobe, PT08 has a lesion involving a portion of the parietal lobe, including areas as the supramarginal gyrus, angular gyrus and parietal lobe. PT06 and 02 showed a very large lesion involving in the former a widespread network of areas of the fronto-parieto-temporal lobes, the insula and the putamen and the right temporo-parietal lobe in the latter.

### MRI Image acquisition and preprocessing

Scanning took place in a 1.5 Tesla Philips scanner at the Borgo Roma Hospital in Verona, using a 15-channels head coil. A whole brain high-resolution (1x1x1 mm^3^) 3D T1-weighted image with magnetization-prepared rapid acquisition gradient echo (MPRAGE) was acquired for all patients to locate the lesion and to allow the registration of functional data together with the anatomical image of each patient. A resting state dataset was acquired covering the whole brain by recording from slices parallel to the bi-commissural line with subjects instructed to lay down with eyes open trying not to think about anything and to stay still as much as possible. This ensures a higher spontaneous activity in occipital attentional regions than in sensorimotor areas [68] and a high reliability in the visual network [69]. One hundred sixty volumes were acquired with TR = 2500ms, Echo Time = 50ms; 30 slices with slice thickness of 4mm, Field of View = 224x224mm, time duration = 6.47m and echo train length = 39. Raw data is available at the link http://hdl.handle.net/2445/136617. Structural T1-weighted as well as functional images were reviewed to identify any possible abnormality such as scanner spikes, incorrect orientation or poor contrast, before inclusion in the statistical analysis. No structural abnormalities other than the presence of the lesion were found.

Structural and functional images were pre-processed using FSL (FMRIB Software Library v5.0) with a pre-processing pipeline adapted from Diez et al.(2015) [70] with parameters adjusted to fit the experimental data. The structural image was reoriented to match the template and a resampled AC-PC aligned image with 6 degrees of freedom was created. The non-brain tissue was removed, and the resulted brain mask was used to segment the brain. The segmented and parcelled brain was finally co-registered to the Montreal Neurological Institute (MNI) Reference Brain [71] using FMRIB linear Image Registration Tool (FLIRT) function. For functional images, slice time correction was performed using the specific slices acquisition order (ascending) to obtain 30 contiguous slices in the AC-PC plane. The resulting images were reoriented to match the template. Motion correction was applied by co-registering all the volumes to the central one, in order to visualize the brain movements along the three axes (x,y,z). Motion statistics as DVARS, Framewise Displacement and Jenkinson’s Framewise Displacement [72] were calculated. The non-brain tissue was removed using the BET function and all volumes were smoothed with a 6-mm FWHM isotropic Gaussian kernel to increase the signal-to-noise ratio. The resulted signal intensity was normalized and a band pass filtering between 0.01 and 0.08 Hz was applied to fit with the main focus of interest in the resting state analysis, i.e. fluctuations of low-frequency signal. Finally, the resulting functional images were registered and normalized to the MNI standard space and the effects of both white matter and cerebrospinal fluid were removed.

### Data analysis: Graph theory-based connectivity analysis

One of the most compelling ways to efficiently illustrate connectivity data sets is by characterizing them as networks defined as a set of neural elements (nodes) and relations among them (edges). In a macroscale brain network, nodes can be defined as regions of interest (ROIs) derived from anatomical atlases and edges as temporal correlations among nodes. Graph theory-based connectivity analyses, as a form of graph-based connectivity modelling, represent a useful way to analyse the strength of functional connections and to assess group differences (for a review see [73]).

#### Regions of interest (ROI) estimation

A commonly used partitioning scheme is atlas-based and uses standard coordinates or anatomical landmarks on the cortical surface to divide the Brain into regions of interest (ROI). In this study ROIs were defined using the Automatic Anatomical Labelling (AAL) atlas [74] that contains 90 cortical and subcortical ROIs, 45 for each hemisphere, that are alternatively interspersed (see Table 3). The mean time-series approach was used to extract time-courses from all ROIs. As mentioned in the Introduction, we assessed the functional connectivity extracted from four RSNs and from the whole brain. The VN was composed by bilateral striate and extrastriate visual areas; the DMN included the hippocampus, the parahippocampal, fusiform and angular gyrus, the precuneus and the MTG; the AN included the frontal eye fields, MFG, IFG, Superior Parietal Lobes, IPL and STG; the SN was composed by the prefrontal cortex, the insula and the cingulate cortex.

**Table 3:** List of the 90 regions that compose the AAL Atlas.

Labels	Abbr.	X	Y	Z
1. Precentral_L	PreCG.L	-38.65	-5.68	50.94
2. Precentral_R	PreCG.R	41.37	-8.21	52.09
3. Frontal_Sup_L	SFGdor.L	-18.45	34.81	42.2
4. Frontal_Sup_R	SFGdor.R	21.9	31.12	43.82
5. Frontal_Sup_Orb_L	ORBsup.L	-16.56	47.32	-13.31
6. Frontal_Sup_Orb_R	ORBsup.R	18.49	48.1	-14.02
7. Frontal_Mid_L	MFG.L	-33.43	32.73	35.46
8. Frontal_Mid_R	MFG.R	37.59	33.06	34.04
9. Frontal_Mid_Orb_L	ORBmid.L	-30.65	50.43	-9.62
10. Frontal_Mid_Orb_R	ORBmid.R	33.18	52.59	-10.73
11. Frontal_Inf_Oper_L	IFGoperc.L	-48.43	12.73	19.02
12. Frontal_Inf_Oper_R	IFGoperc.R	50.2	14.98	21.41
13. Frontal_Inf_Tri_L	IFGtriang.L	-45.58	29.91	13.99
14. Frontal_Inf_Tri_R	IFGtriang.R	50.33	30.16	14.17
15. Frontal_Inf_Orb_L	ORBinf.L	-35.98	30.71	-12.11
16. Frontal_Inf_Orb_R	ORBinf.R	41.22	32.23	-11.91
17. Rolandic_Oper_L	ROL.L	-47.16	-8.48	13.95
18. Rolandic_Oper_R	ROL.R	52.65	-6.25	14.63
19. Supp_Motor_Area_L	SMA.L	-5.32	4.85	61.38
20. Supp_Motor_Area_R	SMA.R	8.62	0.17	61.85
21. Olfactory_L	OLF.L	-8.06	15.05	-11.46
22. Olfactory_R	OLF.R	10.43	15.91	-11.26
23. Frontal_Sup_Medial_L	SFGmed.L	-4.8	49.17	30.89
24. Frontal_Sup_Medial_R	SFGmed.R	9.1	50.84	30.22
25. Frontal_Med_Orb_L	ORBsupmed.L	-5.17	54.06	-7.4
26. Frontal_Med_Orb_R	ORBsupmed.R	8.16	51.67	-7.13
27. Rectus_L	REC.L	-5.08	37.07	-18.14
28. Rectus_R	REC.R	8.35	35.64	-18.04
29, Insula_L	INS.L	-35.13	6.65	3.44
30. Insula_R	INS.R	39.02	6.25	2.08
31. Cingulum_Ant_L	ACG.L	-4.04	35.4	13.95
32. Cingulum_Ant_R	ACG.R	8.46	37.01	15.84
33. Cingulum_Mid_L	DCG.L	-5.48	-14.92	41.57
34. Cingulum_Mid_R	DCG.R	8.02	-8.83	39.79
35. Cingulum_Post_L	PCG.L	-4.85	-42.92	24.67
36. Cingulum_Post_R	PCG.R	7.44	-41.81	21.87
37. Hippocampus_L	HIP.L	-25.03	-20.74	-10.13
38. Hippocampus_R	HIP.R	29.23	-19.78	-10.33
39. ParaHippocampal_L	PHG.L	-21.17	-15.95	-20.7
40. ParaHippocampal_R	PHG.R	25.38	-15.15	-20.47
41. Amygdala_L	AMYG.L	-23.27	-0.67	-17.14
42. Amygdala_R	AMYG.R	27.32	0.64	-17.5
43. Calcarine_L	CAL.L	-7.14	-78.67	6.44
44. Calcarine_R	CAL.R	15.99	-73.15	9.4
45. Cuneus_L	CUN.L	-5.93	-80.13	27.22
46. Cuneus_R	CUN.R	13.51	-79.36	28.23
47. Lingual_L	LING.L	-14.62	-67.56	-4.63
48. Lingual_R	LING.R	16.29	-66.93	-3.87
49. Occipital_Sup_L	SOG.L	-16.54	-84.26	28.17
50. Occipital_Sup_R	SOG.R	24.29	-80.85	30.59
51. Occipital_Mid_L	MOG.L	-32.39	-80.73	16.11
52. Occipital_Mid_R	MOG.R	37.39	-79.7	19.42
53. Occipital_Inf_L	IOG.L	-36.36	-78.29	-7.84
54. Occipital_Inf_R	IOG.R	38.16	-81.99	-7.61
55. Fusiform_L	FFG.L	-31.16	-40.3	-20.23
56. Fusiform_R	FFG.R	33.97	-39.1	-20.18
57. Postcentral_L	PoCG.L	-42.46	-22.63	48.92
58. Postcentral_R	PoCG.R	41.43	-25.49	52.55
59. Parietal_Sup_L	SPG.L	-23.45	-59.56	58.96
60. Parietal_Sup_R	SPG.R	26.11	-59.18	62.06
61. Parietal_Inf_L	IPL.L	-42.8	-45.82	46.74
62. Parietal_Inf_R	IPL.R	46.46	-46.29	49.54
63. SupraMarginal_L	SMG.L	-55.79	-33.64	30.45
64. SupraMarginal_R	SMG.R	57.61	-31.5	34.48
65. Angular_L	ANG.L	-44.14	-60.82	35.59
66. Angular_R	ANG.R	45.51	-59.98	38.63
67. Precuneus_L	PCUN.L	-7.24	-56.07	48.01
68. Precuneus_R	PCUN.R	9.98	-56.05	43.77
69. Paracentral_Lobule_L	PCL.L	-7.63	-25.36	70.07
70. Paracentral_Lobule_R	PCL.R	7.48	-31.59	68.09
71. Caudate_L	CAU.L	-11.46	11	9.24
72. Caudate_R	CAU.R	14.84	12.07	9.42
73. Putamen_L	PUT.L	-23.91	3.86	2.4
74. Putamen_R	PUT.R	27.78	4.91	2.46
75. Pallidum_L	PAL.L	-17.75	-0.03	0.21
76. Pallidum_R	PAL.R	21.2	0.18	0.23
77. Thalamus_L	THA.L	-10.85	-17.56	7.98
78. Thalamus_R	THA.R	13	-17.55	8.09
79. Heschl_L	HES.L	-41.99	-18.88	9.98
80. Heschl_R	HES.R	45.86	-17.15	10.41
81. Temporal_Sup_L	STG.L	-53.16	-20.68	7.13
82. Temporal_Sup_R	STG.R	58.15	-21.78	6.8
83. Temporal_Pole_Sup_L	TPOsup.L	-39.88	15.14	-20.18
84. Temporal_Pole_Sup_R	TPOsup.R	48.25	14.75	-16.86
85. Temporal_Mid_L	MTG.L	-55.52	-33.8	-2.2
86. Temporal_Mid_R	MTG.R	57.47	-37.23	-1.47
87. Temporal_Pole_Mid_L	TPOmid.L	-36.32	14.59	-34.08
88. Temporal_Pole_Mid_R	TPOmid.R	44.22	14.55	-32.23
89. Temporal_Inf_L	ITG.L	-49.77	-28.05	-23.17
90. Temporal_Inf_R	ITG.R	53.69	-31.07	-22.32

Labels and abbreviation of the 90 regions composing the AAL Atlas. The last three columns represent the MNI coordinates of the centre of each region of interest.

#### Network matrix

The simplest way to investigate similarities between two regions of interest is by looking at their time-series correlation using Pearson’s correlation coefficient (“full correlation” approach). The Pearson Correlation is a matrix of N x N dimensions, where N = 90 (number of ROIs) and each value of the matrix represents the strength of correlation coefficient between the time-series of two ROIs. The autocorrelations and the anti-correlations were set to zero. Indeed, according to the literature, the neural basis of negative correlations during rest remains a topic under debate and can be related to neurobiological information as well as to pre-processing artifact or noise [75]. Moreover, negative coefficients extracted were close to 0 in both groups indicating the absence of a significant effect. Correlation coefficients were transformed to Z-statistics using Fisher’s r-to-Z transformation before performing graph theory analysis. We created correlation matrices considering both the whole brain and the RSNs mentioned in the introduction separately.

#### Graph theory analysis

Graph theoretic techniques can provide useful information by extracting higher level summary measures from network matrix to describe aspects of network functioning. Therefore, adjacent weighted undirected matrices were created by thresholding the correlation coefficients to graphically represent density and intensity of functional links on the cortical surface, maintaining the magnitudes of correlational interactions, using the software BrainNet Viewer (http://www.nitrc.org/projects/bnv/) [76]. The application of a threshold to the correlation matrices is fundamental to delete weak correlation coefficients that could represent spurious connections without biological relevance [77] and alter the topology of the network. Indeed, it has been demonstrated that the inclusion of low random connections possibly representing false positive can lead to a high probability of modifying the network metrics in terms of increase of global efficiency and decrease of network clustering. Considering that our clinical population showed a general lower overall functional connectivity, we decided not to apply a proportional threshold that could bring to the inclusion of spurious connections to maintain a fixed network cost, increasing the possibility of including false positive (error type I), that have demonstrated to be more detrimental than false negative (error type II) to the computation of network metrics [78,79]. For all these reasons, we chose to apply a range of absolute thresholds (r = 0.4–0.5 in steps of 0.01), that would allow to minimize the bias in the threshold selection and to control for error types I and II. Adjacent unweighted undirected matrices were created maintaining information on the presence or absence of functional connections, by binarizing the thresholded adjacent weighted matrix of both groups separately, as described by Rubinov and Sporns in 2010 [80]. These matrices were used first of all to visualize the topology of the brain network and then to extract graph theoretic summary measures to assess significant differences in the functional network structure using the Brain Connectivity Toolbox of Matlab [80]. Graph metrics included indices of functional integration as the characteristic path length (the average shortest path length between all possible pairs of nodes in a network) and the global efficiency (the reciprocal of the harmonic mean of the path length) as well as indices of functional segregation as the nodal clustering coefficient (normalized number of pairs of each node’s neighbors that are connected with each other) and local efficiency (index of integration of a node with its immediate neighbors). Nodal metrics included the node degree which indicates the number of edges connecting one node with all the others (for a detailed description of the graph theory analysis see [81,82]). Once the graph measures were extracted, we evaluated the statistical difference between groups comparing values of clustering coefficient, local efficiency and node degree extracted from each node of the group adjacent binary matrices. Then we applied the Wilcoxon rank test for independent samples, where each row represented the graph value extracted from a specific node. FDR was applied to correct for multiple comparisons. Moreover, we extracted the average characteristic path length and the mean global efficiency from each group matrix to describe the main difference in the functional integration of the group network.

#### Functional segregation

Functional segregation assumes that brain regions have the ability to develop specialized processing tasks and then integrate all the information into complex processing stages [20]. It defines the ability for specialized processes to involve densely interconnected groups of brain regions arranged within modules or clusters. The network matrix can be used to infer the intensity of the ROIs’ signal correlation. Moreover, if we consider each pairwise correlation coefficient as the side of a triangle connecting 3 edges represented by 3 ROIs, we can compute the area of the triangle between neighbouring ROIs. The result represents an index of the intensity of pairwise correlations among ROIs, meaning that it would be an index of the strength of functional connectivity. The areas of the cycles can be calculated using Heron’s expression, which is:

$$A=\sqrt{s(s-a)(s-b)(s-c)}$$

Where a, b and c are the side lengths of the triangle and s is the semi perimeter, which is:

$$s=\frac{\left(a+b+c\right)}{2}$$

The areas of all possible triangles were calculated and a threshold represented by the area of an hypothetical triangle whose sides were *r*_*xy*_ > 0.6, was applied to each 3-cycle triangles, in order to assess only the most representative functional networks. This criterion allows the reduction of type I error since the selected threshold implies a significant value of p<0.0001. Its validity has been already demonstrated by similar data reported by Farràs-Permanyer et al., 2019 [83]. In the results, bigger triangles indicate a more intense functional correlation among nodes (ROI’s). The frequency of significant triangles and their mean area can be used as an informative tool to evaluate the strength of functional connectivity. Therefore, we extracted statistical estimators of triangles (number, mean, median, skeweness, std and range) to give a more precise description of functional connectivity in each group. The Wilcoxon rank test for independent samples was applied to highlight group differences in the frequency distribution of 3-cycles triangles and the Chi-Square Test for given probabilities was applied to evaluate differences in the number of significant triangles.

## Results

**Fig 2:**
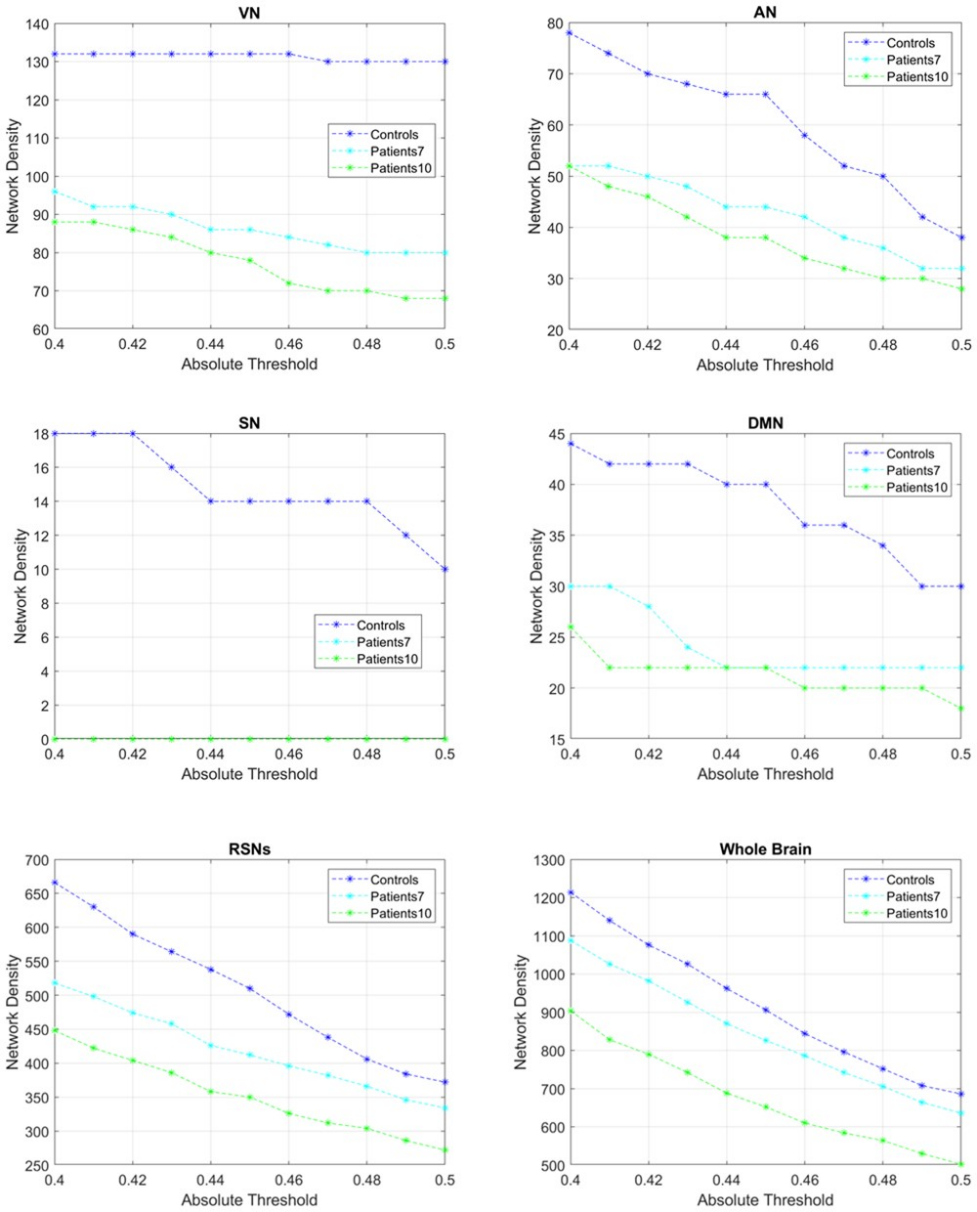
Sensitivity analysis of networks’ density. Sensitivity analysis showing changes in network density according to the absolute threshold applied to the pairwise correlation coefficients, within the range of r = 0.4–0.5, considering intra- and inter-network as well as the whole brain functional connectivity. VN = Visual Network; DMN = Default Mode Network; AN = Attentional Network; SN = Salience Network; RSNs = Resting State Networks.

The trend of networks’ density with different absolute thresholds (Fig 2) showed that the difference tended to reduce when applying the highest absolute threshold (r = 0.5), mainly when considering the Attentional and Default Mode Networks, as well as the inter-networks and the whole brain functional connectivity. For this reason, in addition to the attempt to reduce the inclusion of false positive and the exclusion of false negative, we decided to extract correlation coefficients higher than 0.5 from the group adjacent weighted undirected matrix and to represent the above threshold connections on the cortical surface in the anatomical space.

Then, the “spring” topology-based layout [84] of the qgraph package for R [85] was applied on adjacent unweighted undirected matrices to evaluate the structure of the system and to represent the architecture behind the connectivity. This kind of force-directed layout allows to visualize the distance between nodes according to their attractive and repulsive forces through an iterative algorithm that leads the network to its stable equilibrium. The Brain Connectivity Toolbox of Matlab was used to extract graph summary measures from adjacent unweighted undirected matrices created for each group, considering the whole brain as well as each RSN separately (for the visualization of the trend of graph measures extracted according to the range of absolute threshold, see S1–S5 Figs). Finally, in order to highlight group differences in functional segregation, the 3-cycle regions with edges, i.e. correlation coefficients, over the 0.6 threshold were plotted to obtain the frequency distribution of significant triangles extracted from pairwise Pearson correlation matrices. Because of the heterogeneity of our experimental group, due to the high variability in lesion site and extent, we present results considering both the entire group of 10 patients and a subgroup of 7 patients, created excluding patients with the biggest lesions involving areas not belonging to the occipital lobe (PT02, PT06, PT08).

### Intra-network connectivity

**Fig 3:**
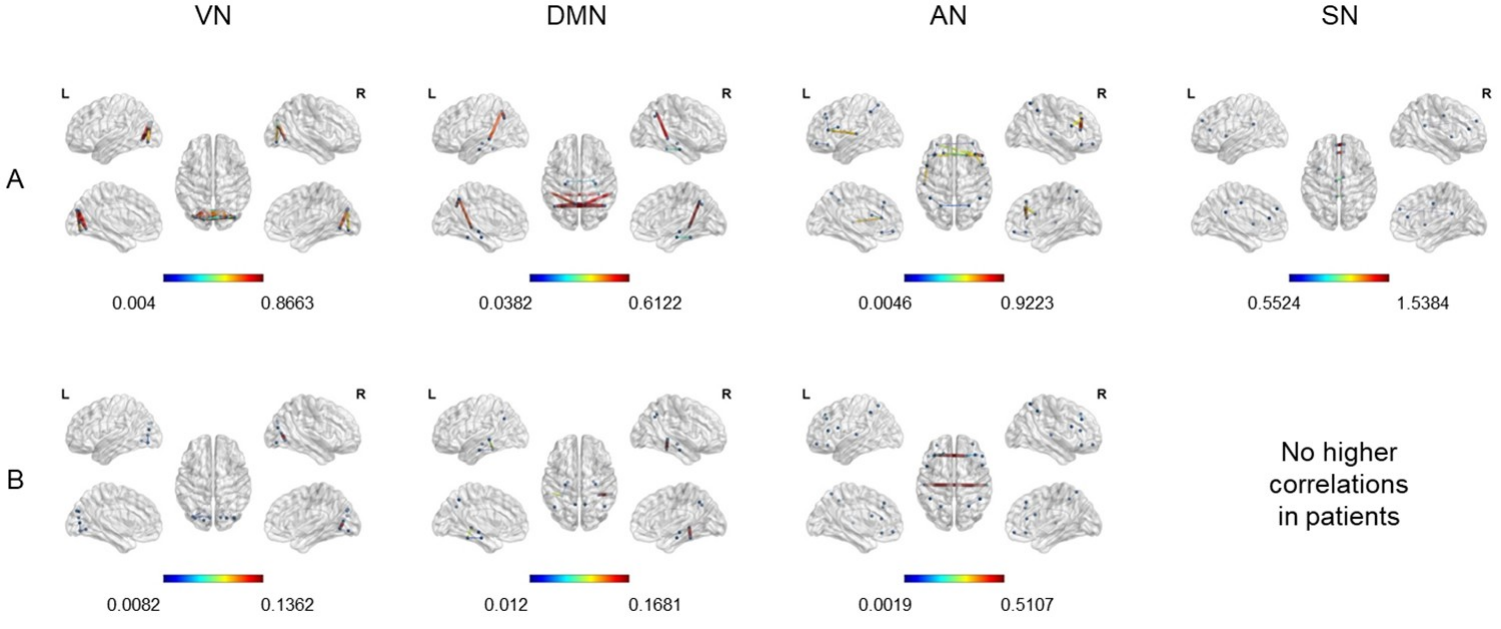
Correlation coefficients higher in controls (A) or in the group of 10 patients (B). Cortical surface representation of correlation coefficients extracted from the threshold (r>0.5) adjacent weighted undirected matrix, higher in controls than patients and vice versa, considering each RSN separately. A: Higher connections in controls than patients. B: Higher connections in the group of 10 patients than controls. VN = Visual Network; DMN = Default Mode Network; AN = Attentional Network; SN = Salience Network.

**Fig 4:**
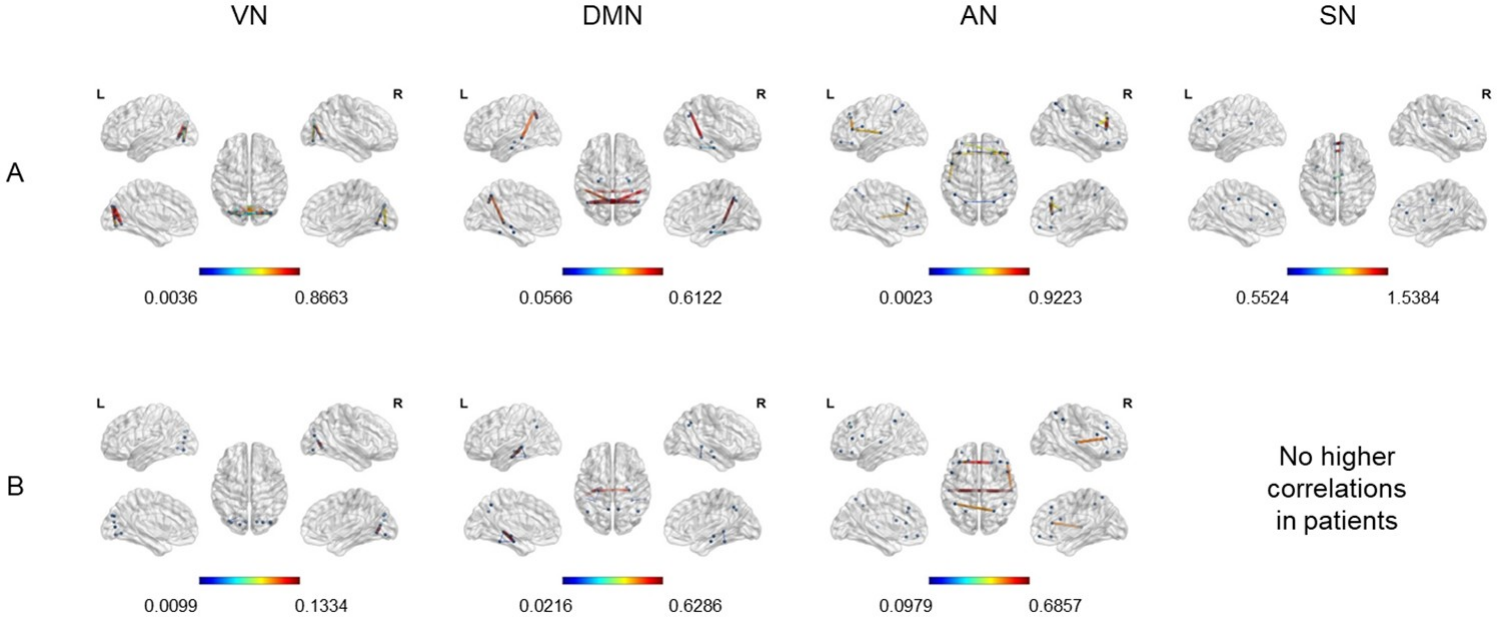
Correlation coefficients higher in controls (A) or in the group of 7 patients (B). Cortical surface representation of correlation coefficients extracted from the threshold (r>0.5) adjacent weighted undirected matrix, higher in controls than patients and vice versa, considering each RSN separately. A: Higher connections in controls than patients. B: Higher connections in the group of 7 patients than controls. VN = Visual Network; DMN = Default Mode Network; AN = Attentional Network; SN = Salience Network.

First of all, we assessed the intra-network connectivity to visualize the main between-group differences within each network. Therefore, we extracted the correlation coefficients higher in controls than patients and vice versa, separately for each RSN (see Figs 3 and 4).

Considering each RS Network separately, we found a gradual decrease in the density and intensity of functional connections in patients compared to healthy controls, with a higher number of above threshold connections in controls (VN = 130; DMN = 30; AN = 38; SN = 10), and gradually lower values in groups of 7 (VN = 80; DMN = 22; AN = 32; SN = no connections) and 10 patients (VN = 68; DMN = 18; AN = 28; SN = no connections).

Considering the **Visual Network**, we found numerous more intense intra- and inter-hemispheric connections between almost all visual areas in controls with respect to both groups of patients. Nevertheless, few slightly higher connections were found in both groups of patients with respect to controls, involving short-range connections between left occipital inferior and calcarine cortex, right calcarine and lingual gyrus and bilateral occipital inferior and lingual gyrus. Concerning the **Attentional Network**, we found higher intra- and inter-hemispheric connections in controls with respect to patients, connecting homologous regions in different hemispheres as well as areas close to each other in the same hemisphere. Inter-hemispheric connections higher in the group of 7 patients with respect to controls can be found, connecting left and right STG as well as left STG and right MFG.

For what concerns the **Salience Network**, we observed some spared higher inter-hemispheric connections in controls with respect to patients, mainly between left and right superior medial frontal gyrus, anterior, median and posterior cingulate.

Finally, regarding the **DMN**, in controls we found stronger inter-hemispheric connections between corresponding areas in right and left hemisphere (parahippocampal, fusiform, angular gyrus and precuneus) and intra-hemispheric connections mainly between bilateral MTG and precuneus, right angular gryus and precuneus, right parahippocampal and fusiform gyrus, left hippocampus and parahippocampal gyrus. In contrast, both groups of patients showed some spared higher intra-hemispheric connections (i.e. fusiform gyrus and MTG), whilst only the group of 7 patients showed higher inter-hemispheric connections between left and right hippocampus.

**Fig 5:**
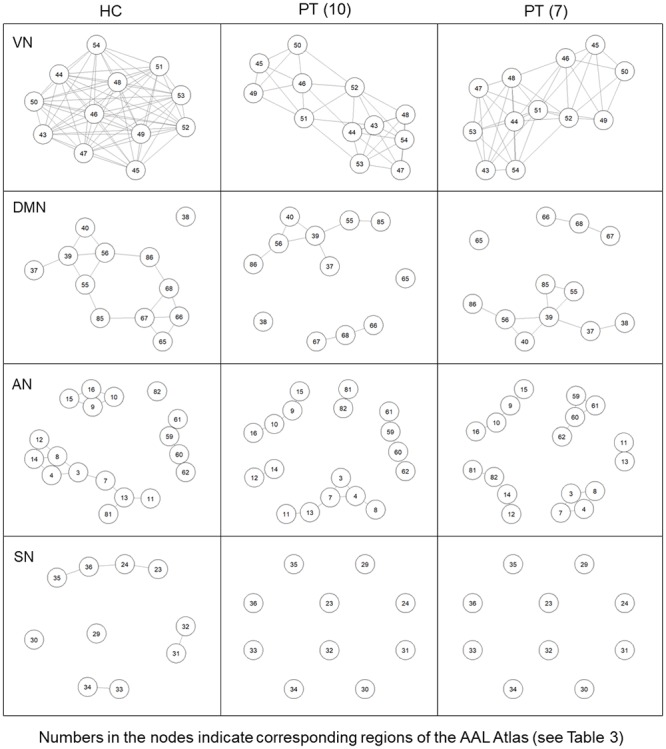
“Spring” topology-based layout in different RSNs separately. RSNs “spring” topology-based layout used to represent the correlation coefficients extracted from the adjacent unweighted undirected matrix (r>0.5), in the group of controls (HC), in the group of 10 patients (PT10) and in the group of 7 patients (PT7). SN = Salience Network; DMN = Default Mode Network; AN = Attentional Network; VN = Visual Network.

The visualization of RS network topology (Fig 5) highlighted differences in the architecture of each resting state network comparing both groups of patients with respect to controls. This difference was higher when considering the VN and consisted of a gradual decrease in the number of connections among nodes, with a more distributed structure and less interconnected nodes in patients. The extreme case was represented by the Salience Network, where no connections among nodes could be extracted from the binary matrices of both groups of patients.

From the adjacent binary matrix of each group and RSN, we extracted the graph measures of functional segregation and integration, to assess differences between controls and clinical population. They confirmed that the main differences was in the **visual network.** Indeed, we extracted significant lower values of node degree (FDR-corrected p<0.001, Z = 4.31), clustering coefficient (FDR-corrected p<0.001, Z = 3.63) and local efficiency (FDR-corrected p<0.001, Z = 3.63) in the group of 10 patients, indicating lower functional segregation and lower number of highly-connected *hubs*. When excluding patients with the biggest lesions, we observed the same trend with a significant difference only in the number of hubs (FDR-corrected p<0.001, Z = 4.23), which was maintained significantly lower in patients than in controls. In both groups of patients we observed lower values of global efficiency and a longer characteristic path length, indicating a lower capacity to integrate information using shortest path routing (Table 4). Considering values extracted from each node, we observed that in each node belonging to the visual network contributed to the mean values extracted (no zero values), with lower node degrees in the superior occipital gyrus and lower clustering coefficient in the middle occipital gyrus (S1 Table) in both groups of patients.

**Table 4:** Local and global measures: Visual Network.

GROUP	CLUSTERING COEFFICIENT	LOCAL EFFICIENCY	CHARACTERISTIC PATH LENGTH	GLOBAL EFFICIENCY	NODE DEGREE
PATIENTS (7)	Mean = 0.83 Std = 0.16	Mean = 0.9147 Std = 0.0838	1.44	0.79	Mean = 6.67 Std = 1.67
PATIENTS (10)	Mean = 0.716 Std = 0.18	Mean = 0.84 Std = 0.1059	1.61	0.74	Mean = 5.67 Std = 1.15
CONTROLS	Mean = 0.98 Std = 0.0071	Mean = 0.99 Std = 0.0035	1.015	0.99	Mean = 10.83 Std = 0.39

Visual Network. Graph measures extracted from the threshold adjacent unweighted matrices (r>0.5) of the group of controls, of 10 patients and of 7 patients. Mean and standard deviation (std) are shown for clustering coefficient, local efficiency and node degree. The average characteristic path length and the index of global efficiency are shown as single values extracted from the group matrix.

In the Attentional Network we observed a decrease in the number of interconnected hubs (FDR-corrected p = 0.0708, Z = 2.26) when comparing the group of 10 patients with respect to controls. All nodes contribute to the mean values (see S2 Table) as no zero values were reported. We extracted lower values of functional segregation, even if this difference was not significant, due to the fact that only few nodes created weak links with pairs of neighbors. Instead, we observed an opposite pattern in the group of 7 patients, where we extracted higher values of functional segregation with respect to controls. Indeed, in this group of patients we extracted more numerous high values of clustering coefficients from those nodes that actually had links (see S2 Table). The characteristic path length was shorter in both groups of patients and the global efficiency was higher indicating a surprisingly higher capacity to integrate information using shortest path routing within this network in patients. This trend was maintained even when excluding patients with the biggest lesion, indicating that the higher functional integration was not directly related to the lesion size (see Table 5).

**Table 5:** Local and global measures: Attentional Network.

GROUP	CLUSTERING COEFFICIENT	LOCAL EFFICIENCY	PATH LENGTH	GLOBAL EFFICIENCY	NODE DEGREE
PATIENTS (7)	Mean = 0.81 Std = 0.26	Mean = 0.86 Std = 0.24	1.44	0.81	Mean = 1.78 Std = 0.73
PATIENTS (10)	Mean = 0.56 Std = 0.38	Mean = 0.56 Std = 0.38	1.76	0.7	Mean = 1.56 Std = 0.7
CONTROLS	Mean = 0.72 Std = 0.24	Mean = 0.82 Std = 0.21	2.25	0.62	Mean = 2.23 Std = 0.9

Attentional Network. Graph measures extracted from the threshold adjacent unweighted matrices (r>0.5) of the group of controls, of 10 patients and of 7 patients. Mean and standard deviation (std) are shown for clustering coefficient, local efficiency and node degree. The average characteristic path length and the index of global efficiency are shown as single values extracted from the group matrix.

In the Salience Network, we could not extract any graph measures in both groups of patients, reflecting the topology of the RS Network, composed only by isolated nodes in patients and by very few connections between pairs of nodes in controls (Fig 5).

Considering the DMN, we observed a lower number of interconnected nodes in the group of patients (FDR-corrected p = 0.145, Z = 1.97). All nodes contribute to the mean values, except for the left angular gyrus that showed no connections with other nodes (see S3 Table). The characteristic path length was shorter in both groups of patients and the global efficiency was higher, indicating a higher level of functional integration, faster information transfer and a minimization of the metabolic cost associating with routing. Moreover, in the group of 10 patients we observed lower values of functional segregation whilst in the group of 7 patients we found the inverse trend, with higher values of functional segregation in patients with respect to controls (see Table 6). Indeed, in both groups of patients we observed a higher number of nodes with no relationships with pairs of neighbors (zero values) but in the group of 7 patients we extracted more numerous high values of clustering coefficients from those nodes that actually had links.

**Table 6:** Local and global measures: Default Mode Network.

GROUP	CLUSTERING COEFFICIENT	LOCAL EFFICIENCY	PATH LENGTH	GLOBAL EFFICIENCY	NODE DEGREE
PATIENTS (7)	Mean = 0.71 Std = 0.4	Mean = 0.71 Std = 0.4	1.93	0.63	Mean = 2 Std = 1.18
PATIENTS (10)	Mean = 0.5 Std = 0.44	Mean = 0.5 Std = 0.44	1.92	0.64	Mean = 1.8 Std = 1.03
CONTROLS	Mean = 0.56 Std = 0.3	Mean = 0.59 Std = 0.3	2.38	0.55	Mean = 2.72 Std = 1

Default Mode Network. Graph measures extracted from the threshold adjacent unweighted matrices (r>0.5) of the group of controls, of 10 patients and of 7 patients. Mean and standard deviation (std) are shown for clustering coefficient, local efficiency and node degree. The average characteristic path length and the index of global efficiency are shown as single values extracted from the group matrix.

### Inter-network connectivity

**Fig 6:**
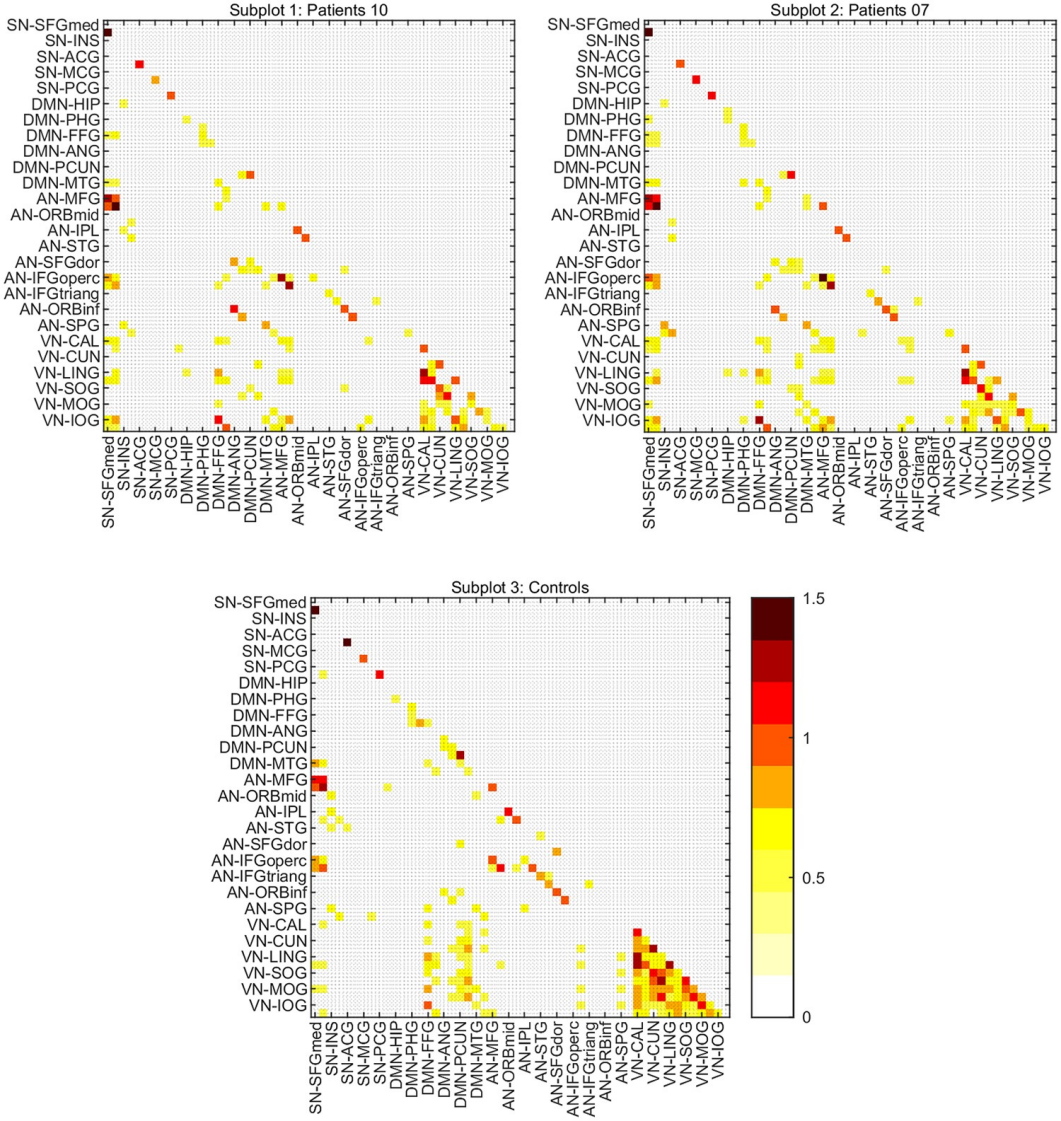
Matrices of correlation coefficients including regions belonging to all RSNs, showing intra- and inter-network correlations. Adjacent weighted undirected correlation matrices (r>0.5) of controls, of 10 patients and of 7 patients, representing intra- and inter-network connectivity. A greater number of positive between-networks correlations was observed in the group of controls. SN (Salience Network): Superior Medial Frontal Cortex, Insula, Anterior, Middle and Posterior Cingulate. DMN (Default Mode Network): Hippocampus, Parahippocampal Gyrus, Fusiform Gyrus, Angular Gyrus, Precuneus, Middle Temporal Cortex. AN (Attentional Network): Middle Frontal Gyrus, Middle Frontal Gyrus Orbital Part, Inferior Parietal, Superior Temporal Gyrus, Superior Frontal Gyrus (dorsolateral), Inferior Frontal Gyrus (opercular, triangular and orbital part), Superior Parietal Gyrus. VN (Visual Network): Calcarine, Cuneus, Lingual Cortex, Superior, Middle and Inferior Occipital Cortex.

Comparing the density and intensity of functional correlations extracted from threshold adjacent weighted matrices between different networks, we found a decrease in inter-network connectivity in both groups of patients, as shown in Fig 6 representing intra- and inter-networks correlation matrices in controls, in the group of 10 patients and in the group of 7 patients (Fig 6).

A greater number of positive connections was observed in controls with respect to patients mainly between the DMN and the VN, mainly between the fusiform gyrus, the precuneus and visual areas. To further analyse the density and intensity of inter-network connectivity across groups, we visualized correlation coefficients higher in controls than in patients and vice versa, considering both the four RSNs previously separately analysed, and the whole brain.

**Fig 7:**
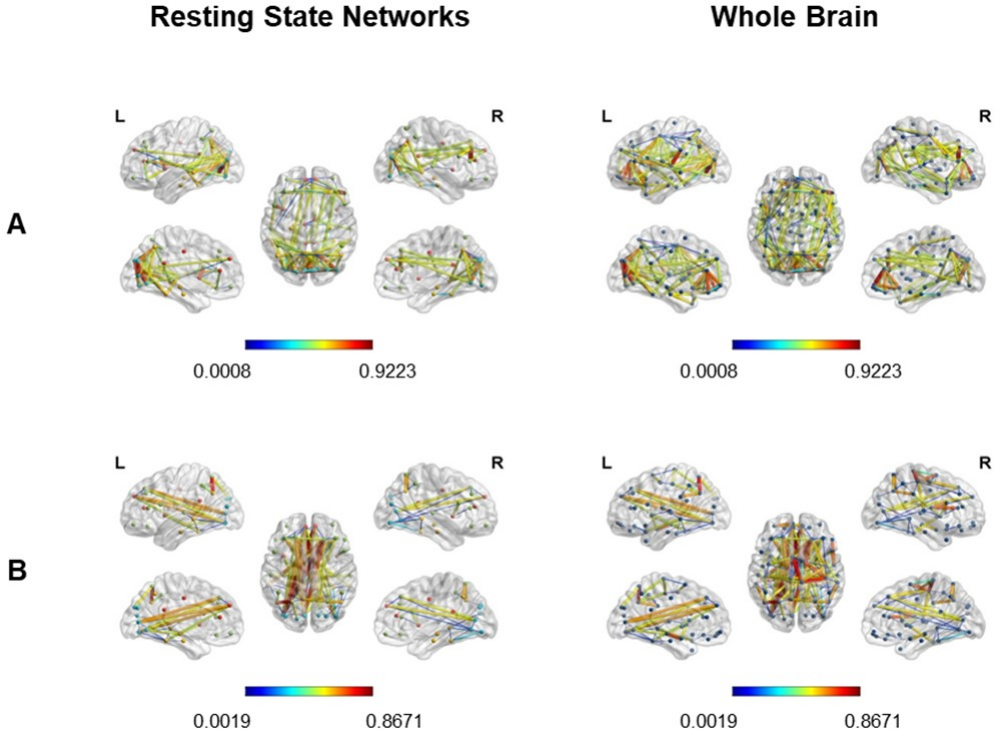
Correlation coefficients higher in controls (A) and in the group of 10 patients (B). Cortical surface representation of the correlation coefficients extracted from the threshold (r>0.5) adjacent weighted matrix, higher in controls than patients and vice versa, considering all RSNs together (left) and the whole brain (right). A: Higher connections in controls. B: Higher connections in the group of 10 patients. Red nodes indicate the Salience Network; Yellow nodes indicate the Default Mode Network; Green nodes indicate the Attentional Network; Blue nodes indicate the Visual Network.

**Fig 8:**
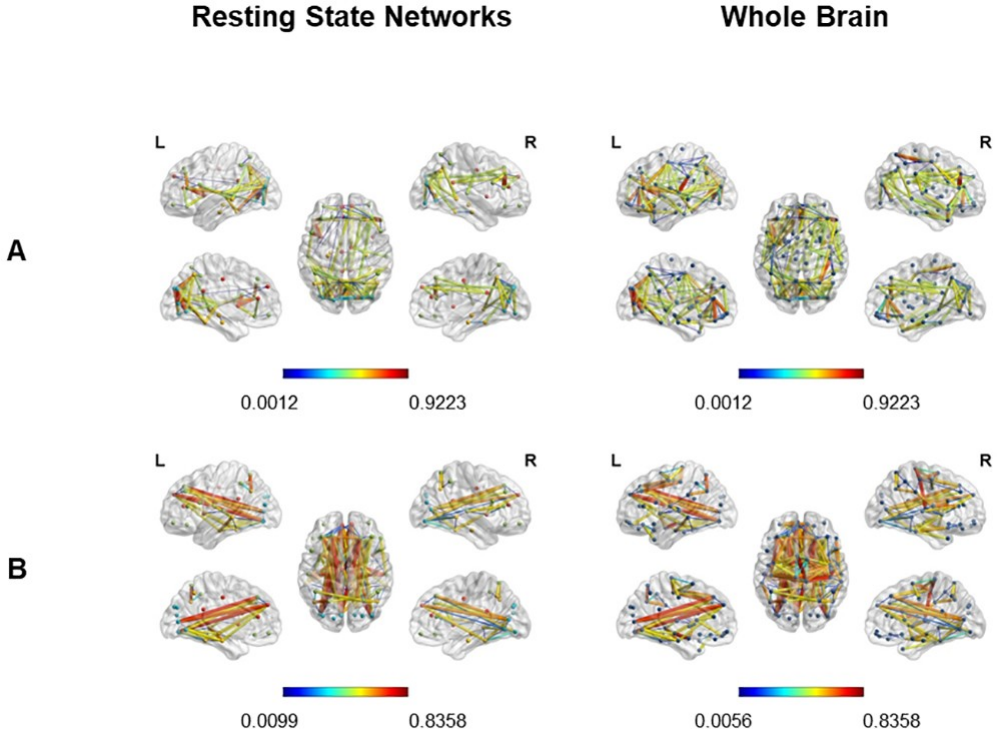
Correlation coefficients higher in controls (A) and in the group of 7 patients (B). Cortical surface representation of the correlation coefficients extracted from the threshold (r>0.5) adjacent weighted matrix, higher in controls than patients and vice versa, considering all RSNs together (left) and the whole brain (right). A: Higher connections in controls. B: Higher connections in the group of 7 patients. Red nodes indicate the Salience Network; Yellow nodes indicate the Default Mode Network; Green nodes indicate the Attentional Network; Blue nodes indicate the Visual Network.

Concerning the RSNs, the representation onto the cortical surface showed a gradual decrease in the density and intensity of functional connections in patients compared to healthy controls, with the number of above threshold connections going from 372 in the group of controls, to 334 in the group of 7 patients, to 272 in the group of 10 patients. This reduction was mainly affecting intra- and inter-hemispheric connections among visual areas (see Figs 7 and 8, left). Moreover, we observed a gradual decrease in inter-hemispheric connections among homotopic areas and among regions close to each other, belonging to the same network (intra-network connectivity) or to different networks (between-network connectivity). Interestingly, we observed an increase in long-range connections mainly between occipital and frontal regions, thus connecting the inferior occipital gyrus, calcarine cortex and lingual gyrus with the frontal superior (Attentional Network) and frontal superior medial (Salience Network) areas.

Also when considering the Whole Brain (see Figs 7 and 8, right), the representation onto the cortical surface revealed a general gradual decrease in both density and intensity of functional correlations in damaged brains, with the number of above threshold connections going from 686 in the group of controls, to 636 in the group of 7 patients, to 502 in the group of 10 patients. This reduction was more evident in intra-hemispheric connections mainly in occipital and frontal areas, and in the inter-hemispheric connections mainly between homotopic occipital and frontal areas. Nevertheless, we found some higher intra- and inter-hemispheric connections between areas structurally distant from each other, as in occipito-frontal regions. Moreover, we observed the same trend of differences in both groups of patients. These results may indicate that the inclusion of patients with biggest lesions involving fronto-parieto-temporal areas could modulate the density and intensity of functional correlations without changing the pattern of reorganization of functional connectivity in damaged brains.

**Fig 9:**
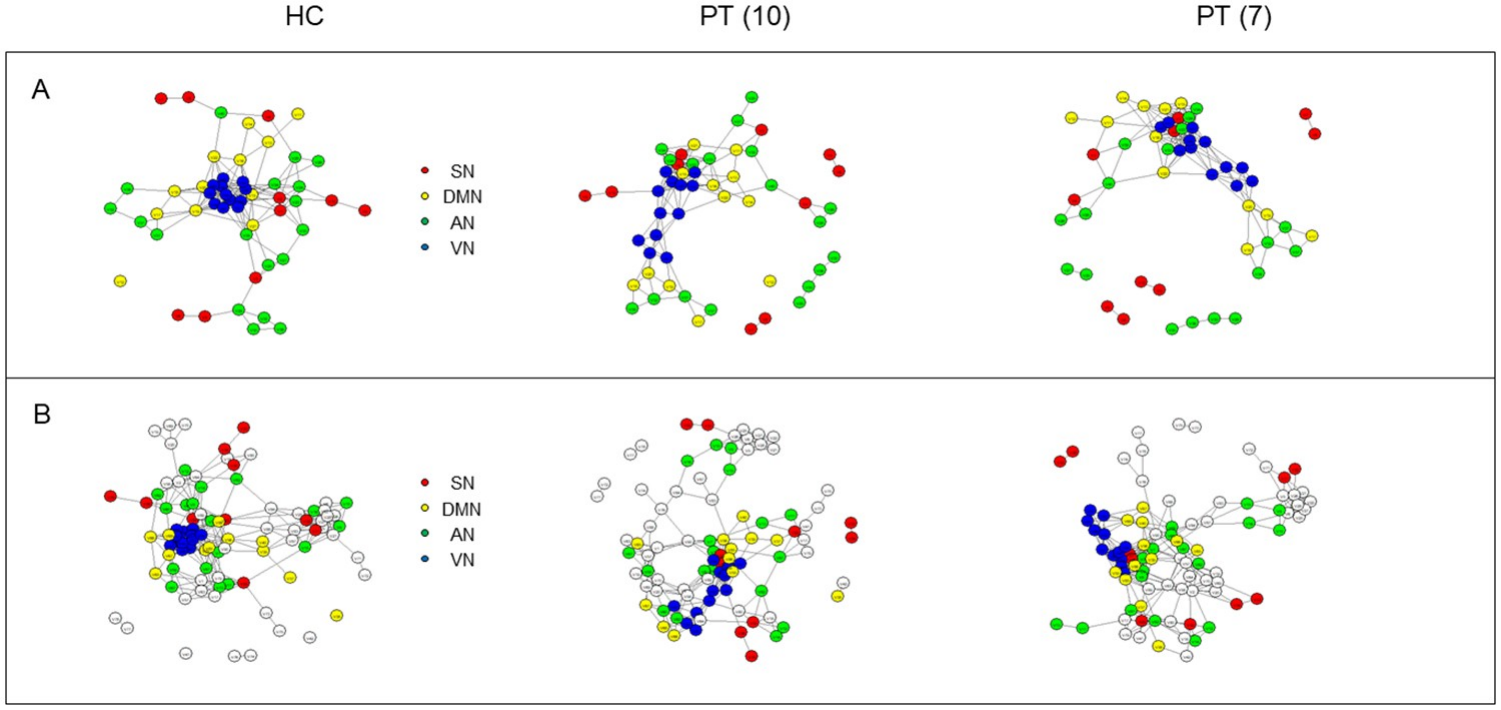
“Spring” topology-based layout in the four RSNs (upper) and in the Whole Brain (lower). “Spring” topology-based layout used to represent the correlation coefficients extracted from the adjacent unweighted undirected matrix of the RSNs (A) and of the whole brain (B), in the group of controls (HC), in the group of 10 patients (PT10) and in the group of 7 patients (PT7). SN = Salience Network; DMN = Default Mode Network; AN = Attentional Network; VN = Visual Network.

The representation of the network architecture revealed the presence of a more compact structure in controls than patients where nodes belonging to the same network are close to each other and organized in well integrated functional clusters (see Fig 9). The less compact structure of patients’ network is quite evident when considering the whole brain of the entire group of patients, where the whole brain structure appeared less specialized, less integrated in terms of interconnected *hubs*, more distributed and less segregated in functional modules (see Fig 9B). Instead, when focusing on the RSNs inter-connectivity, the network appeared more segregated in functional modules composed by areas belonging to different RSNs (see Fig 9A).

These results are confirmed by the extraction of graph summary measures. Indeed, considering the RSNs, we extracted lower number of interconnected *hubs* and higher values of functional segregation in both groups of patients with respect to controls, with a bigger difference when excluding patients with the biggest lesions. Indeed in this case we extracted more numerous high values of clustering coefficient despite the presence of more numerous nodes reporting zero values (see S4 Table). Interestingly, we extracted the shortest characteristic path length and the highest global efficiency in the group of 7 patients, indicating a higher capacity to integrate information using less metabolic cost and shortest path routing (Table 7).

**Table 7:** Local and global measures: RSNs.

GROUP	CLUSTERING COEFFICIENT	LOCAL EFFICIENCY	PATH LENGTH	GLOBAL EFFICIENCY	NODE DEGREE
PATIENTS (7)	Mean = 0.65 Std = 0.21	Mean = 0.77 Std = 0.2	2.83	0.48	Mean = 6.42 Std = 5.07
PATIENTS (10)	Mean = 0.61 Std = 0.24	Mean = 0.73 Std = 0.21	3.22	0.42	Mean = 5.33 Std = 3.9
CONTROLS	Mean = 0.58 Std = 0.2	Mean = 0.7 Std = 0.21	3.29	0.42	Mean = 7.29 Std = 5.52

RSNs. Graph measures extracted from the threshold adjacent unweighted matrices (r>0.5) of the group of controls, of 10 patients and of 7 patients. Mean and standard deviation (std) are shown for clustering coefficient, local efficiency and node degree. The average characteristic path length and the index of global efficiency are shown as single values extracted from the group matrix.

Concerning the whole brain we extracted higher values of functional segregation and integration in controls than in patients with a bigger difference when considering the group of 10 patients, where there was a significantly lower number of interconnected hubs (FDR-corrected p = 0.0029, Z = 3.3). All nodal values contribute to the mean node degree extracted as we did not find nodes with zero values. Moreover, in patients and controls we observed a similar trend in the distribution of nodal clustering coefficients with higher values in controls (see S5 Table). In both groups of patients we observed lower values of global efficiency and a longer characteristic path length, indicating a lower capacity to integrate information using shortest path routing (Table 8).

**Table 8:** Local and global measures: Whole Brain.

GROUP	CLUSTERING COEFFICIENT	LOCAL EFFICIENCY	PATH LENGTH	GLOBAL EFFICIENCY	NODE DEGREE
PATIENTS (7)	Mean = 0.57 Std = 0.2	Mean = 0.71 Std = 0.02	3.79	0.35	Mean = 7.07 Std = 4.5
PATIENTS (10)	Mean = 0.55 Std = 0.22	Mean = 0.67 Std = 0.22	4.08	0.33	Mean = 5.57 Std = 3.59
CONTROLS	Mean = 0.58 Std = 1.9	Mean = 0.72 Std = 0.16	3.25	0.39	Mean = 7.88 Std = 4.71

Whole Brain. Graph measures extracted from the threshold adjacent unweighted matrices (r>0.5) of the group of controls, of 10 patients and of 7 patients. Mean and standard deviation (std) are shown for clustering coefficient, local efficiency and node degree. The average characteristic path length and the index of global efficiency are shown as single values extracted from the group matrix.

### Functional segregation

In Table 9 we report the statistical estimators of the triangles for each RSN separately and for the whole brain, to give a more precise description of functional connectivity in each group. The number of triangles and the skewness/asymmetry of the distribution represent the parameters that show the highest level of group variability. The former indicates an abrupt reduction in the number of triangles in patients, meaning a reduction in the functional segregation mainly in the whole brain and in the visual network; the latter indicates a different asymmetry in the distribution of probabilities mainly in the RSNs. The test highlights a significant group difference in the frequency distribution of 3-cycles triangles in the whole brain (Z = 2.27; p = 0.0234) in the entire group of patients, and in the Salience Network (Z = 2.24, p = 0.02) when excluding patients with the biggest lesions. The number of triangles was not homogeneous between controls and the group of 10 patients (Whole brain: χ^2^ = 57.09; DF = 1, p<0.001; VN: χ^2^ = 86.09; DF = 1, p<0.001) with the former showing a significant increase in the overall counts.

**Table 9:** Statistical estimators of the triangles between ROIs.

GROUP	N triangles	MEAN	MEDIAN	SKEWNESS	SD	MIN	MAX
HC_VN	**138**	0.193	0.183	1.2642	0.0331	0.1559	0.3161
PT(10)_VN	**21**	0.198	0.19	0.81	0.0036	0.1579	0.28
PT(7)_VN	**21**	0.19	0.2	0.7	0.03	0.16	0.26
HC_DMN	**2**	0.16	0.16	0	0.0074	0.16	0.17
PT(10)_DMN	**0**						
PT(7)_DMN	**2**	0.16	0.16	0	0.0057	0.16	0.16
HC_AN	**12**	0.169	0.16	1.19	0.0149	0.157	0.198
PT(10)_AN	**11**	0.168	0.17	0.11	0.008	0.156	0.18
PT(7)_AN	**13**	0.174	0.17	0.82	0.011	0.157	0.2
HC_SN	**9**	0.18	0.179	0.78	0.0129	0.168	0.21
PT(10)_SN	**4**	0.167	0.17	-0.71	0.0081	0.156	0.17
PT(7)_SN	**10**	0.167	0.163	0.77	0.009	0.157	0.18
HC_WB	**944**	0.1759	0.1698	2.6657	0.0213	0.1559	0.3161
PT(10)_WB	**643**	0.1735	0.1681	2.79	0.0185	0.1559	0.2997
PT(07)_WB	**870**	0.175	0.17	2.51	0.02	0.16	0.32

Number of significant triangles extracted from the correlation matrices of controls (HC), of the group of 10 patients (PT10) and of the group of 7 patients (PT7). Statistical estimators are extracted from each group and RSN as well as from the whole brain: mean, median, skewness, standard deviation (SD) and range.

**Fig 10:**
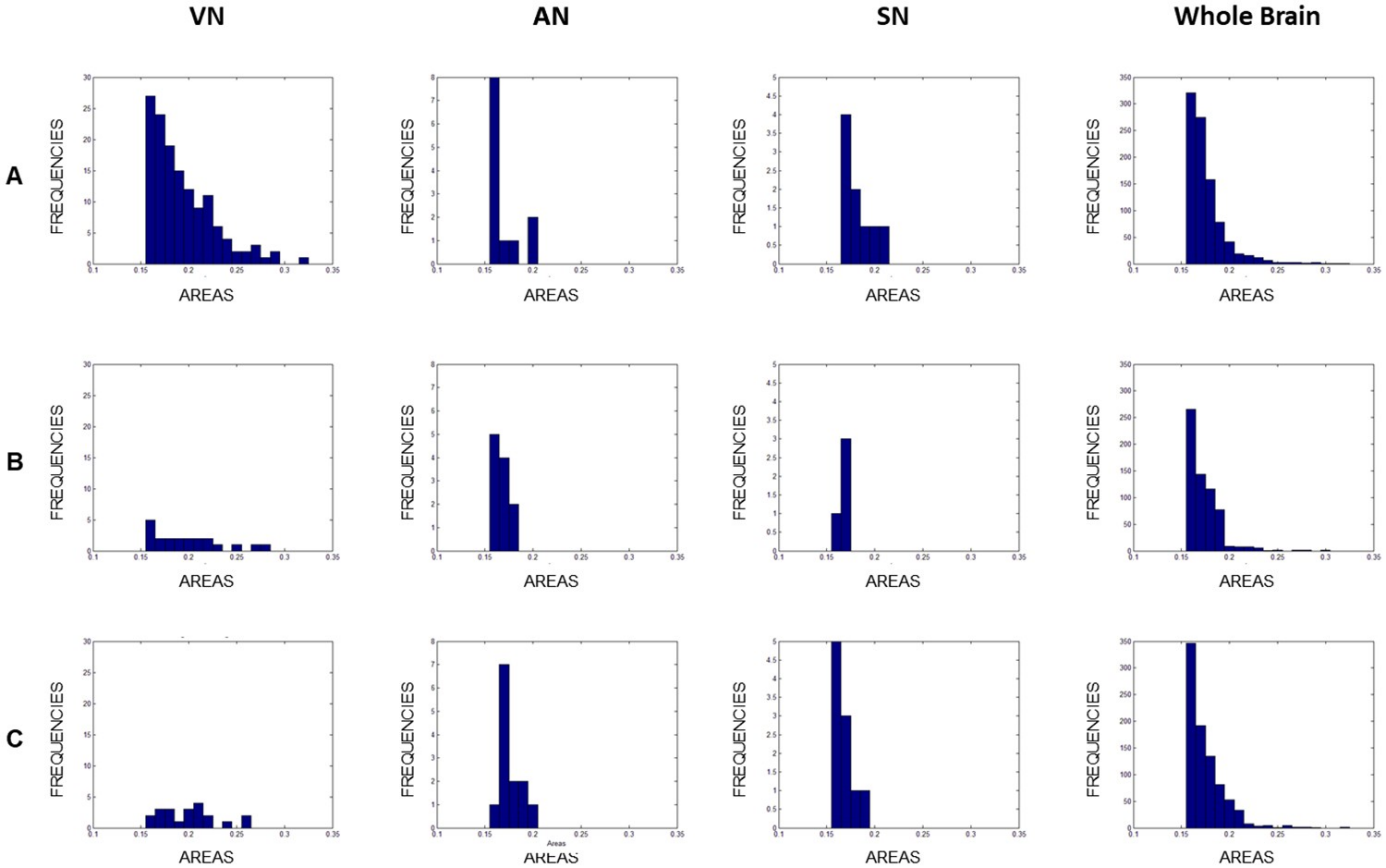
3-cycle triangles in the VN, AN, SN and in the whole brain. Representation of 3-cycle triangles area distribution in the group of controls (A), of 10 patients (B) and of 7 patients (C), for the VN, AN, SN and the whole brain.

Furthermore, we extracted the triangles’ area distribution to assess for differences between the two groups. At first glance, they show a similar tendency in triangle’s area distribution when considering the whole brain; in contrast, a clear difference emerges when considering resting state networks (Fig 10) and mainly the VN.

Taken together, these results suggest that damaged brains are characterized by a reorganization of the whole cerebral network expressed by a general decrease in density and intensity of functional correlations, in the functional segregation and integration, with a more distributed architecture characterized by less segregated and poorly integrated functional nodes. This is more evident for the whole brain and the VN. Indeed, this subnetwork shows a significant reduction in functional segregation and integration, regardless of the lesion extent, demonstrating to be the subnetwork most affected by the lesion. Surprisingly, we observe a higher global efficiency in the DMN and in the AN that indicates a more efficient integration of information between nodes, independent from the lesion extent, and that may indicate a functional reorganization of the brain following the injury. Moreover, looking at the inter-network connectivity, we observe an increase in functional specialization mainly of DMN and VN (lower between-network connectivity) and in functional segregation, that is reflected in a less compact structure, highly organized in functional clusters.

## Discussion

The present research provides new insight on the functional connectivity network in hemianopic patients with different lesion sites and extents compared with a group of age-matched healthy participants. Objectives of the study were two-fold: First, to extract and compare the functional connectivity pattern by means of a full correlation analysis. Second, to assess functional intra- and inter-network characteristics focusing mainly on modular integration and segregation defined as the strengthening of within-network, weakening of between-network connectivity and enhancement of global integration. Brain networks have been shown to have a clear modular structure, that is some groups of nodes are strongly connected to each other and weakly connected to other modules. This modular, yet integrated, topology may enable both functional specialization and coordination across modules potentially reducing interference among systems and therefore facilitating cognitive performance. High signal coherence within a network renders its subcomponents more functionally coupled resulting in a greater functional specialization [86]. The assessment of differences in these parameters during RS represents the basis of a more complete understanding of differences in brain activation and behavioural performance in task-related paradigms [49].

As to the first objective, the full correlation analysis revealed a general decrease in brain synchronous activation in patients when extracting correlation coefficients among regions of interest, thus demonstrating the involvement of a widespread network belonging not only to the visual system.

As to the second objective, we applied network-based tools to describe the architecture of functional connections with a graph representation that reflects the network’s functions for both whole brain and different subnetworks separately.

As expected, the **VN** is the network where the main difference was observed with a general decrease in the intra-network connectivity for both intra- and inter-hemispheric connections. These results indicate a significant decrease in functional specialization of the visual system. Significant lower levels of functional segregation and integration were extracted indicating an abrupt impairment of its functioning, independent from lesion extent. Kim and colleagues (2019) [87] linked the increase of inter-hemispheric connections within the VN with visual field recovery in post-stroke patients with hemianopia, by performing serial RS fMRI, within 1 week and at 1 or 3 months after ischemic stroke in the visual cortex. Therefore, they highlighted the importance of inter-hemispheric intra-network connectivity as crucial for visual field rehabilitation. Furthermore, a reduction of intra-network functional connectivity within the VN was observed in a group of patients with acute optic neuritis [88]. Moreover, in patients with primary angle-closure glaucoma (PACG), Chen and colleagues (2019) found a decrease in the short-range functional connectivity density, mainly in visual and visual-associated areas thus confirming the alteration of spontaneous activity in the visual cortex [89]. In contrast, no alteration in the inter-hemispheric functional connectivity within visual cortices was found in patients with visuospatial impairment (neglect), in whom a breakdown of functional connectivity in fronto-parietal networks was reported [90].

Considering the **Attentional Fronto-Parietal Network**, we observed a general decrease in the inter-hemispheric connections between homotopic areas, despite some spared inter-hemispheric connections that were stronger in patients than controls, mainly connecting left and right STG as well as left STG and right MFG. Moreover, we found a lower mean node degree, reflecting the topology of the network composed by nodes organized in linear clusters, with only one connection between pairs of nodes. Interestingly, the results showed an inverse trend in functional segregation in the two groups of patients that was lower than controls when considering all patients and higher when excluding patients where these areas were actually lesioned. Conversely, we found an increase in functional integration in both groups of patients indicating that the capacity to integrate information was independent from lesion extent. Concerning the **Salience Network**, usually involved in filtering salient stimuli as well as in the detection and integration of sensory stimuli, we observed few higher inter-hemispheric connections in controls than in patients, as a consequence of the fact that only few strong connections survived after applying the absolute threshold. Moreover, we could not extract any connection between pairs of nodes. This result confirms the SN topology, composed only by isolated nodes. This alteration in functional connectivity was independent from the lesion extent as was found even in patients whose lesions did not directly damage this network. Alteration at the level of the salience network have been linked to the impairment of both cognition and self-monitoring, compromising the ability to detect salient external and internal events and causing different kinds of psychopathologies, such as anxiety disorders, schizophrenia, drug addiction and pain [91,92].

As to the **DMN**, our results indicate a general decrease in patients of the intra-network connectivity involving both intra and inter-hemispheric connections between homotopic areas, with some spared stronger intra-hemispheric short-range connections mainly between the MTG and the fusiform gyrus. These results reveal a decrease in the functional specialization as a consequence of the reduction of signal coherence within the subnetwork. Importantly, when considering the DMN in the subgroup of patients with the smallest lesion we observed some spared stronger long-range inter-hemispheric connections mainly between left and right hippocampus. The graph measures at the same time indicated a decrease in the number of interconnected *hubs* and an increase in functional integration, with higher efficiency in the capacity to integrate information, regardless of the lesion extent. Instead, functional segregation was affected by lesion extent being lower than in controls when considering the entire sample but higher when considering the subgroup of patients with the smallest lesion. Alterations of DMN functional connectivity have been reported in normal brain aging as well as in patients with various disorders. Indeed, a decrease in intra-network functional connectivity within the DMN has been also demonstrated in normal brain aging [93,94] generally associated with poor executive function, memory and processing speed [95] and reflecting a progressive loss of functional specialization within brain networks related to higher cognitive functions [96]. Furthermore, functional alterations in the DMN have been reported in patients with Alzheimer and mild cognitive impairment [97,98], with a decrease in within-network connectivity, and in patients with major depression [99], with an increase in within-network connectivity. The decreased coupling between DMN and VN observed in hemianopic patients regardless of the lesion extent, reflects an increase in segregation between networks and can be attributed to a neuroplastic mechanism that likely depends on the intricate balance between intra-network connectivity reduction (functional specialization) and inter-network coupling (between network segregation). Probably, the inverse reduced segregation between DMN (task-negative ICN) and task-positive networks might be the cause of the decreased regulation in normal brain aging [100] or of the reduced functioning in many psychiatric disorders [101,102]. A similar non adaptive, widespread, increased internetworks synchrony, probably due to an impaired inhibitory circuitry, has been observed also in subjects with Down syndrome [103]. Our results are not in keeping with the increased connectivity reported by Boucard et al., 2016 [104] between two visual networks and the inferior posterior DMN in a blind patient with degeneration of the visual pathway, probably underlying their patient’s vivid visual imagery in absence of any visual input. However, the difference in the type of visual impairment can explain the different results indicating a more adaptive compensative mechanism activated in hemianopic patients whose vision is still preserved in a portion of the visual field.

In the assessment of RSN inter-connectivity and of the whole-brain functional connectivity in patients we found stronger long-range connections mainly between the occipital and frontal lobe of both hemispheres despite a decrease in the short-range intra-hemisphere and inter-hemispheric connections between homotopic regions, which may play an important role in maintaining a balance between excitation and inhibition across hemispheres [105]. It is relevant to mention that this pattern of changes is independent from the lesion extent, as it was present in both groups of patients. Interestingly, when considering the RSNs inter-connectivity, we extracted higher values of functional segregation and integration in both groups of patients indicating a neuroplastic mechanism possibly activated to compensate for the reduced intra-network connectivity and the decreased density and intensity of connections. Instead, the architecture of the whole brain appeared less segregated, compact and integrated in terms of number of interconnected nodes (*hubs*) when considering the entire group of patients. The graph measures confirmed the presence of a less segregated (lower functional segregation) and integrated (lower nodal degree) network with lower values of modular segregation and less numerous inter-connected hubs, regardless of lesion extent. These results suggest that the presence of the lesion may determine the impairment of a distributed network involving not only visual areas.

Finally, the study of functional segregation by means of the 3-cycles triangles confirmed the reduction of functional segregation in hemianopic patients when considering the whole brain and the VN.

Taken together these results suggest that during the rest period the whole brain functioning of patients is different from that of healthy controls, with higher long-range correlation across distant regions in different lobes despite the general decrease in density and intensity of connections, likely suggesting an attempt to compensate for the general synchronization loss caused by the lesion.

A strong point of this study is represented by the population studied. As mentioned before, the assessment of functional connectivity with fMRI in patients with a retro-chiasmatic lesion is a novel enterprise. Few studies have been published, mainly using electroencephalography, but, to our knowledge, very few fMRI studies focused on the assessment of task-independent functional connectivity in this specific clinical population, mainly considering the functional connectivity within the VN and not the whole brain system [87]. Furthermore, the assessment of brain activity without an active task enabled us to avoid one of the main problems emerging when performing cognitive tasks with hemianopic patients, namely the maintenance of the fixation on the central point to avoid the displacement of the blind hemifield and guarantee the location of the stimulus in the blind hemifield. Indeed, unlike the assessment of visual stimulation related brain activity, the assessment of RS functional connectivity with eyes open takes into account the possibility of spontaneously moving eyes in both populations, without carrying the confounding effect produced by the shift of focal vision and the consequent increase of visual awareness as no visual stimulus is shown. Importantly, we used 3-cycle regions to complement the information extracted from correlation matrices and density maps. This method is widely used in functional connectivity research because it greatly helps in the identification of intra- and inter-network connections [106].

There are some limitations to be considered in this study. First, the sample size is quite small and not homogeneous as brain damage is somewhat different in location and size. Second, a technical problem of RS fMRI protocols is represented by the possible confounding of automatic bodily motion or cardiac pulsations [107,108]. It has been reported that subject’s movements can modulate functional connectivity determining artefactual increased local and reduced long-range connectivity [72]. Moreover, the threshold applied to the 3-cycle segregation analysis was selected to highlight triangles whose side was greater than r = 0.6. However, further research could improve the optimization of this intensity threshold. Concerning the results, we have to highlight that our conclusions depend mainly on descriptive results that could be speculative and vulnerable to outliers included in the sample as well as influenced by the difference in networks’ density among groups (as a consequence of the application of an absolute threshold).

## Conclusions

In conclusion, the present study provides new insight on the functional connectivity network in a specific group of chronic patients characterized by visual impairment caused by ischemic or haemorrhagic stroke. Our results show a significant alteration in the intrinsic architecture of a large-scale brain system determining a general decrease in the whole brain intra- and inter-hemispheric functional connectivity that goes beyond a significant impairment of synchronous activation in the visual network. These results are in broad agreement with those by Quigley et al., 2001 [109] in stroke patients where they found that large lesions affecting a distributed major neural network may decrease functional connectivity. Similar mechanisms have been described in different clinical populations as in MS (multiple-sclerosis) patients with a significant reduction in within-network coherence and modular segregation [110]. Interestingly, in our patients the main impairment was located in the visual network confirming prior investigations revealing that specific behavioural deficits are often associated with decreased within-task related network but increased within-task unrelated networks connectivity [111]. Future work needs to address the assessment of within-task unrelated networks connectivity and extend these findings to hemianopic patients. In spite of this general decrease in synchronization one can find some spared higher long-range correlations between regions belonging to different modules, a decrease in inter-network connectivity mainly between DMN and VN and an increase in the capacity to integrate information using shortest path routing within the DMN and the AN, regardless of the size of the injury. These results may represent underlying mechanisms of neuroplasticity consequent to an injury, operating to compensate the general reduced functional connectivity.

## Supporting information

S1 Table Node degrees (ND) and clustering coefficients (CC) extracted from each node of the Visual Network. (PDF)

S2 Table Node degrees (ND) and clustering coefficients (CC) extracted from each node of the Attentional Network. (PDF)

S3 Table Node degrees (ND) and clustering coefficients (CC) extracted from each node of the Default Mode Network. (PDF)

S4 Table Node degrees (ND) and clustering coefficients (CC) extracted from the RSNs analysed. (PDF)

S5 Table Node degrees (ND) and clustering coefficients (CC) extracted from each node of the whole brain. (PDF)

S1 Fig Sensitivity analysis of graph measures in the Visual Network (VN). Sensitivity analysis showing changes in the graph measures extracted from the adjacent unweighted undirected matrices of the Visual Network (VN), according to the absolute threshold applied to the pairwise correlation coefficients, within the range r = 0.4–0.5.
(TIF)

S2 Fig Sensitivity analysis of graph measures in the Attentional Network (AN). Sensitivity analysis showing changes in the graph measures extracted from the adjacent unweighted undirected matrices of the Attentional Network (AN), according to the absolute threshold applied to the pairwise correlation coefficients, within the range r = 0.4–0.5.
(TIF)

S3 Fig Sensitivity analysis of graph measures in the Default Mode Network (DMN). Sensitivity analysis showing changes in the graph measures extracted from the adjacent unweighted undirected matrices of the Default Mode Network (DMN), according to the absolute threshold applied to the pairwise correlation coefficients, within the range r = 0.4–0.5.
(TIF)

S4 Fig Sensitivity analysis of graph measured in the Resting State Networks (RSNs). Sensitivity analysis showing changes in the graph measures extracted from the adjacent unweighted undirected matrices considering the inter-network functional connectivity, according to the absolute threshold applied to the pairwise correlation coefficients, within the range r = 0.4–0.5.
(TIF)

S5 Fig Sensitivity analysis of graph measures in the whole brain. Sensitivity analysis showing changes in the graph measures extracted from the adjacent unweighted undirected matrices considering whole brain functional connectivity, according to the absolute threshold applied to the pairwise correlation coefficients, within the range r = 0.4–0.5.
(TIF)
