# Supplementary material for: Functional interactions in patients with hemianopia: A graph theory-based connectivity study of resting fMRI signal
Source: PLoS One. 2020 Jan 6;15(1):e0226816. doi: 10.1371/journal.pone.0226816 (PMC6944357; doi:10.1371/journal.pone.0226816)
Supplement: S1 Table — (PDF) [file pone.0226816.s001.pdf]

VN	HC_ND	PT(10)_ND	PT(7)_ND	HC_CC	PT(10)_CC	PT(7)_CC
CAL.L	11	7	6	0.98	0.71	1.00
CAL.R	11	7	8	0.98	0.67	0.75
CUN.L	10	4	5	1.00	0.83	0.90
CUN.R	11	6	7	0.98	0.53	0.67
LING.L	11	5	6	0.98	1.00	1.00
LING.R	11	6	8	0.98	0.87	0.75
SOG.L	11	**4**	**5**	0.98	0.83	0.90
SOG.R	11	**4**	**4**	0.98	0.67	1.00
MOG.L	11	6	10	0.98	**0.40**	**0.58**
MOG.R	11	7	8	0.98	**0.48**	**0.61**
IOG.L	11	6	6	0.98	0.73	1.00
IOG.R	10	6	7	1.00	0.87	0.86
