# Supplementary material for: Functional interactions in patients with hemianopia: A graph theory-based connectivity study of resting fMRI signal
Source: PLoS One. 2020 Jan 6;15(1):e0226816. doi: 10.1371/journal.pone.0226816 (PMC6944357; doi:10.1371/journal.pone.0226816)
Supplement: S2 Table — (PDF) [file pone.0226816.s002.pdf]

AN	HC_ND	PT(10)_ND	PT(7)_ND	HC_CC	PT(10)_CC	PT(7)_CC
MFG.L	2	3	2	**0.00**	0.33	1.00
MFG.R	4	1	2	0.50	**0.00**	1.00
ORBmid.L	3	2	2	0.67	**0.00**	**0.00**
ORBmid.R	2	2	2	1.00	**0.00**	**0.00**
IPL.L	1	1	2	**0.00**	**0.00**	1.00
IPL.R	1	1	1	**0.00**	**0.00**	**0.00**
STG.L	1	1	1	**0.00**	**0.00**	**0.00**
STG.R	**0**	1	2	**0.00**	**0.00**	**0.00**
SFGdor.L	3	2	3	0.33	1.00	0.67
SFGdor.R	3	3	3	0.67	0.33	0.67
IFGoperc.L	1	1	1	**0.00**	**0.00**	**0.00**
IFGoperc.R	2	1	1	1.00	**0.00**	**0.00**
IFGtriang.L	3	2	1	**0.00**	**0.00**	**0.00**
IFGtriang.R	3	1	2	0.67	**0.00**	**0.00**
ORBinf.L	2	1	1	1.00	**0.00**	**0.00**
ORBinf.R	3	1	1	0.67	**0.00**	**0.00**
SPG.L	2	2	2	**0.00**	**0.00**	1.00
SPG.R	2	2	3	**0.00**	**0.00**	0.33
