# Supplementary material for: Functional interactions in patients with hemianopia: A graph theory-based connectivity study of resting fMRI signal
Source: PLoS One. 2020 Jan 6;15(1):e0226816. doi: 10.1371/journal.pone.0226816 (PMC6944357; doi:10.1371/journal.pone.0226816)
Supplement: S3 Table — (PDF) [file pone.0226816.s003.pdf]

DMN	HC_ND	PT(10)_ND	PT(7)_ND	HC_CC	PT(10)_CC	PT(7)_CC
HIP.L	1	1	2	**0.00**	**0.00**	**0.00**
HIP.R	**0**	**0**	1	**0.00**	**0.00**	**0.00**
PHG.L	4	4	5	0.33	0.17	0.20
PHG.R	2	2	2	1.00	1.00	1.00
FFG.L	3	2	2	0.33	**0.00**	1.00
FFG.R	4	3	3	0.33	0.33	0.33
ANG.L	2	0	0	1.00	**0.00**	**0.00**
ANG.R	3	1	1	0.67	**0.00**	**0.00**
PCUN.L	4	1	1	0.33	**0.00**	**0.00**
PCUN.R	3	2	2	0.33	**0.00**	**0.00**
MTG.L	2	1	2	**0.00**	**0.00**	1.00
MTG.R	2	1	1	**0.00**	**0.00**	**0.00**
