# Supplementary material for: Functional interactions in patients with hemianopia: A graph theory-based connectivity study of resting fMRI signal
Source: PLoS One. 2020 Jan 6;15(1):e0226816. doi: 10.1371/journal.pone.0226816 (PMC6944357; doi:10.1371/journal.pone.0226816)
Supplement: S4 Table — (PDF) [file pone.0226816.s004.pdf]

| | HC_ND | PT(10)_ND | PT(7)_ND | HC_CC | PT(10)_CC | PT(7)_CC |
|---|---|---|---|---|---|---|
| SFGmed.L | 8 | 11 | 15 | 0.57 | 0.71 | 0.68 |
| SFGmed.R | 12 | 13 | 14 | 0.38 | 0.67 | 0.76 |
| INS.L | 4 | 3 | 3 | 0.33 | 0.00 | 0.33 |
| INS.R | 2 | 3 | 3 | 0.00 | 0.33 | 0.67 |
| ACG.L | 2 | 1 | 1 | 0.00 | 0.00 | 0.00 |
| ACG.R | 1 | 1 | 1 | 0.00 | 0.00 | 0.00 |
| MCG.L | 1 | 1 | 1 | 0.00 | 0.00 | 0.00 |
| MCG.R | 2 | 1 | 1 | 0.00 | 0.00 | 0.00 |
| PCG.L | 1 | 1 | 1 | 0.00 | 0.00 | 0.00 |
| PCG.R | 3 | 2 | 1 | 0.33 | 0.00 | 0.00 |
| HIP.L | 1 | 3 | 4 | 0.00 | 0.00 | 0.17 |
| HIP.R | 0 | 0 | 1 | 0.00 | 0.00 | 0.00 |
| PHG.L | 4 | 4 | 8 | 0.33 | 0.17 | 0.43 |
| PHG.R | 2 | 2 | 2 | 1.00 | 1.00 | 1.00 |
| FFG.L | 11 | 11 | 11 | 0.56 | 0.58 | 0.75 |
| FFG.R | 8 | 8 | 12 | 0.39 | 0.39 | 0.61 |
| ANG.L | 3 | 2 | 2 | 0.67 | 1.00 | 1.00 |
| ANG.R | 7 | 4 | 3 | 0.52 | 0.50 | 0.67 |
| PCUN.L | 14 | 4 | 4 | 0.48 | 0.50 | 0.67 |
| PCUN.R | 13 | 5 | 9 | 0.77 | 0.40 | 0.42 |
| MTG.L | 8 | 7 | 10 | 0.36 | 0.71 | 0.60 |
| MTG.R | 7 | 5 | 7 | 0.48 | 0.40 | 0.57 |
| MFG.L | 6 | 10 | 13 | 0.60 | 0.76 | 0.78 |
| MFG.R | 7 | 12 | 13 | 0.57 | 0.67 | 0.78 |
| ORBmid.L | 3 | 1 | 1 | 0.33 | 0.00 | 0.00 |
| ORBmid.R | 2 | 2 | 2 | 1.00 | 1.00 | 1.00 |
| IPL.L | 4 | 3 | 1 | 0.33 | 0.00 | 0.00 |
| IPL.R | 5 | 2 | 3 | 0.40 | 1.00 | 0.67 |
| STG.L | 4 | 1 | 1 | 0.17 | 0.00 | 0.00 |
| STG.R | 3 | 1 | 1 | 0.67 | 0.00 | 0.00 |
| SFGdor.L | 3 | 5 | 5 | 0.33 | 0.30 | 0.50 |
| SFGdor.R | 2 | 5 | 6 | 0.00 | 0.40 | 0.40 |
| IFGoperc.L | 4 | 8 | 9 | 0.50 | 0.46 | 0.83 |
| IFGoperc.R | 10 | 7 | 10 | 0.44 | 0.95 | 0.89 |
| IFGtriang.L | 3 | 2 | 2 | 0.67 | 0.00 | 0.00 |
| IFGtriang.R | 2 | 2 | 2 | 1.00 | 0.00 | 0.00 |
| ORBinf.L | 3 | 2 | 3 | 0.67 | 1.00 | 0.67 |
| ORBinf.R | 2 | 2 | 2 | 0.00 | 1.00 | 1.00 |
| SPG.L | 8 | 3 | 4 | 0.50 | 0.00 | 0.33 |
| SPG.R | 3 | 3 | 5 | 0.00 | 0.00 | 0.20 |
| CAL.L | 15 | 14 | 14 | 0.76 | 0.57 | 0.77 |
| CAL.R | 13 | 10 | 15 | 0.86 | 0.53 | 0.62 |
| CUN.L | 13 | 4 | 6 | 0.87 | 0.83 | 0.87 |
| CUN.R | 16 | 7 | 8 | 0.68 | 0.43 | 0.64 |
| LING.L | 16 | 10 | 13 | 0.71 | 0.69 | 0.63 |
| LING.R | 18 | 12 | 17 | 0.56 | 0.67 | 0.60 |
| SOG.L | 15 | 6 | 7 | 0.77 | 0.40 | 0.67 |

| | | | | | | |
|---|---|---|---|---|---|---|
| SOG.R | 15 | 6 | 5 | 0.74 | 0.40 | 1.00 |
| MOG.L | 19 | 7 | 13 | 0.57 | 0.38 | 0.50 |
| MOG.R | 15 | 8 | 10 | 0.75 | 0.39 | 0.53 |
| IOG.L | 15 | 13 | 15 | 0.67 | 0.64 | 0.65 |
| IOG.R | 14 | 12 | 14 | 0.62 | 0.58 | 0.69 |
