# Supplementary material for: Functional interactions in patients with hemianopia: A graph theory-based connectivity study of resting fMRI signal
Source: PLoS One. 2020 Jan 6;15(1):e0226816. doi: 10.1371/journal.pone.0226816 (PMC6944357; doi:10.1371/journal.pone.0226816)
Supplement: S5 Table — (PDF) [file pone.0226816.s005.pdf]

	HC_ND	PT(10)_ND	PT(7)_ND	HC_CC	PT(10)_CC	PT(7)_CC
PreCG.L	12	8	12	0.35	0.39	0.36
PreCG.R	9	8	12	0.39	0.43	0.29
SFGdor.L	8	11	16	0.54	0.67	0.60
SFGdor.R	8	15	16	0.61	0.51	0.58
ORBsup.L	11	8	11	0.60	0.50	0.51
ORBsup.R	9	7	10	0.67	0.57	0.58
MFG.L	6	10	13	0.40	0.40	0.56
MFG.R	14	10	13	0.37	0.67	0.63
ORBmid.L	5	5	8	0.40	0.50	0.61
ORBmid.R	4	4	3	0.50	0.33	0.67
IFGoperc.L	5	2	2	0.60	0.00	0.00
IFGoperc.R	3	3	4	1.00	1.00	0.50
IFGtriang.L	8	3	1	0.64	0.00	0.00
IFGtriang.R	8	3	4	0.43	1.00	0.83
ORBinf.L	7	3	4	0.33	0.33	0.33
ORBinf.R	7	2	3	0.29	0.00	0.33
ROL.L	8	4	6	0.68	0.50	0.47
ROL.R	4	5	5	0.67	0.60	0.70
SMA.L	2	4	6	1.00	0.50	0.80
SMA.R	4	5	7	0.33	0.70	0.62
OLF.L	9	4	7	0.61	0.83	0.81
OLF.R	9	4	6	0.75	0.83	1.00
SFGmed.L	10	11	16	0.49	0.71	0.63
SFGmed.R	13	14	15	0.41	0.63	0.70
ORBsupmed.L	9	5	6	0.72	0.80	1.00
ORBsupmed.R	10	8	10	0.73	0.46	0.60
REC.L	11	7	8	0.65	0.67	0.86
REC.R	10	6	8	0.76	0.73	0.86
INS.L	9	7	7	0.47	0.19	0.43
INS.R	4	6	7	0.50	0.40	0.33
ACG.L	11	2	5	0.62	0.00	0.50
ACG.R	13	1	2	0.53	0.00	1.00
DCG.L	1	1	2	0.00	0.00	0.00
DCG.R	3	1	3	0.33	0.00	0.33
PCG.L	1	1	1	0.00	0.00	0.00
PCG.R	3	2	1	0.33	0.00	0.00
HIP.L	1	4	5	0.00	0.17	0.20
HIP.R	0	1	2	0.00	0.00	0.00
PHG.L	7	4	9	0.33	0.17	0.33
PHG.R	3	2	2	1.00	1.00	1.00
AMYG.L	0	2	3	0.00	1.00	0.67
AMYG.R	0	1	2	0.00	0.00	0.00
CAL.L	15	14	14	0.76	0.57	0.77
CAL.R	13	10	15	0.86	0.53	0.62
CUN.L	13	4	6	0.87	0.83	0.87
CUN.R	16	7	8	0.68	0.43	0.64
LING.L	16	10	13	0.71	0.69	0.63

LING.R	18	12	17	0.56	0.67	0.60
SOG.L	15	6	7	0.77	0.40	0.67
SOG.R	15	6	5	0.74	0.40	1.00
MOG.L	20	7	13	0.54	0.38	0.50
MOG.R	15	8	10	0.75	0.39	0.53
IOG.L	16	14	16	0.62	0.60	0.60
IOG.R	15	13	15	0.56	0.54	0.66
FFG.L	13	12	12	0.46	0.56	0.68
FFG.R	10	9	15	0.36	0.42	0.49
PoCG.L	5	8	11	0.80	0.39	0.38
PoCG.R	5	11	10	0.80	0.29	0.33
SPG.L	6	8	8	0.47	0.32	0.32
SPG.R	5	7	7	0.60	0.38	0.38
IPL.L	6	5	7	0.60	0.90	0.52
IPL.R	5	5	5	0.60	0.70	0.60
SMG.L	9	4	6	0.58	1.00	0.40
SMG.R	9	6	6	0.33	0.47	0.40
ANG.L	4	6	4	0.50	0.67	0.83
ANG.R	7	5	4	0.52	0.50	0.67
PCUN.L	14	5	4	0.48	0.30	0.67
PCUN.R	13	5	9	0.77	0.40	0.42
PCL.L	3	6	8	0.67	0.40	0.54
PCL.R	2	6	7	1.00	0.53	0.71
CAU.L	2	1	3	0.00	0.00	0.33
CAU.R	1	1	1	0.00	0.00	0.00
PUT.L	2	2	1	0.00	0.00	0.00
PUT.R	1	1	1	0.00	0.00	0.00
PAL.L	1	1	1	0.00	0.00	0.00
PAL.R	1	2	3	0.00	0.00	0.00
THA.L	1	1	1	0.00	0.00	0.00
THA.R	1	1	2	0.00	0.00	0.00
HES.L	7	3	4	0.62	1.00	1.00
HES.R	3	4	5	1.00	0.50	0.40
STG.L	12	5	7	0.44	0.30	0.38
STG.R	7	5	10	0.29	0.30	0.29
TPOsup.L	9	4	3	0.44	0.00	0.33
TPOsup.R	6	3	5	0.33	0.00	0.30
MTG.L	10	9	11	0.38	0.56	0.56
MTG.R	10	6	8	0.33	0.47	0.54
TPOmid.L	5	1	3	0.70	0.00	0.33
TPOmid.R	8	3	2	0.43	0.33	0.00
ITG.L	11	8	7	0.49	0.71	0.71
ITG.R	6	8	13	0.40	0.39	0.36
