# Supplementary figures and images for: Functional interactions in patients with hemianopia: A graph theory-based connectivity study of resting fMRI signal
Source: PLoS One. 2020 Jan 6;15(1):e0226816. doi: 10.1371/journal.pone.0226816 (PMC6944357; doi:10.1371/journal.pone.0226816)

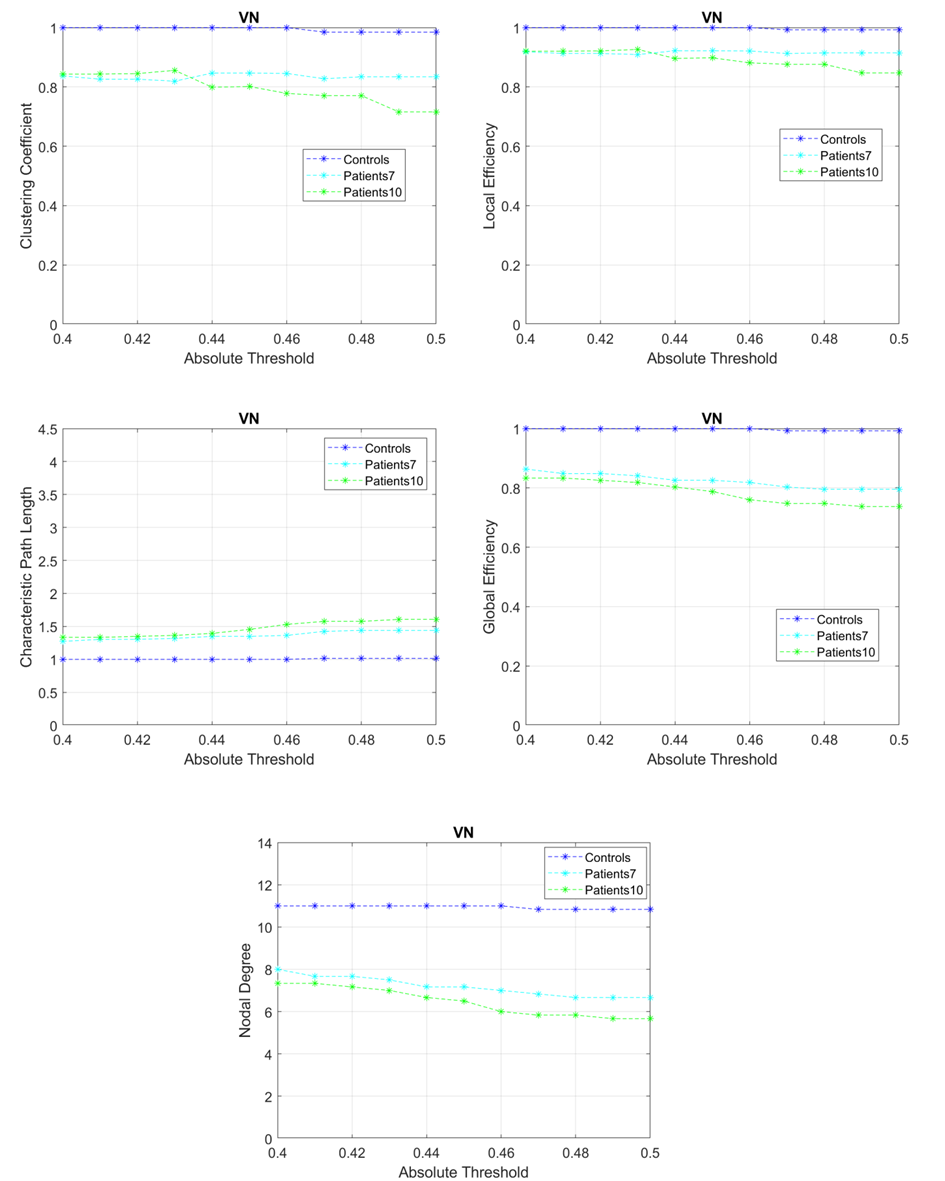

Supplement: S1 Fig — Sensitivity analysis showing changes in the graph measures extracted from the adjacent unweighted undirected matrices of the Visual Network (VN), according to the absolute threshold applied to the pairwise correlation coefficients, within the range r = 0.4–0.5. (TIF) [file pone.0226816.s006.tif]

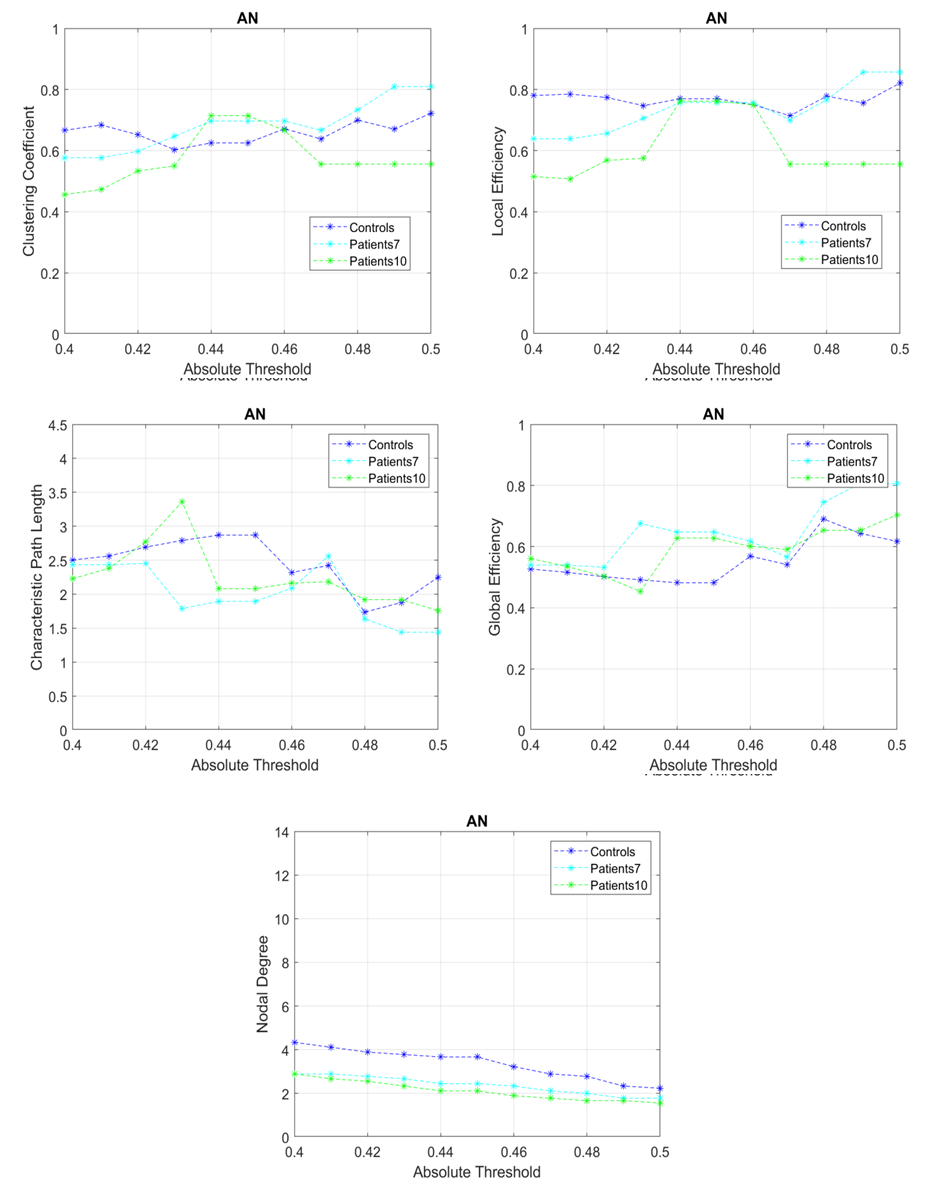

Supplement: S2 Fig — Sensitivity analysis showing changes in the graph measures extracted from the adjacent unweighted undirected matrices of the Attentional Network (AN), according to the absolute threshold applied to the pairwise correlation coefficients, within the range r = 0.4–0.5. (TIF) [file pone.0226816.s007.tif]

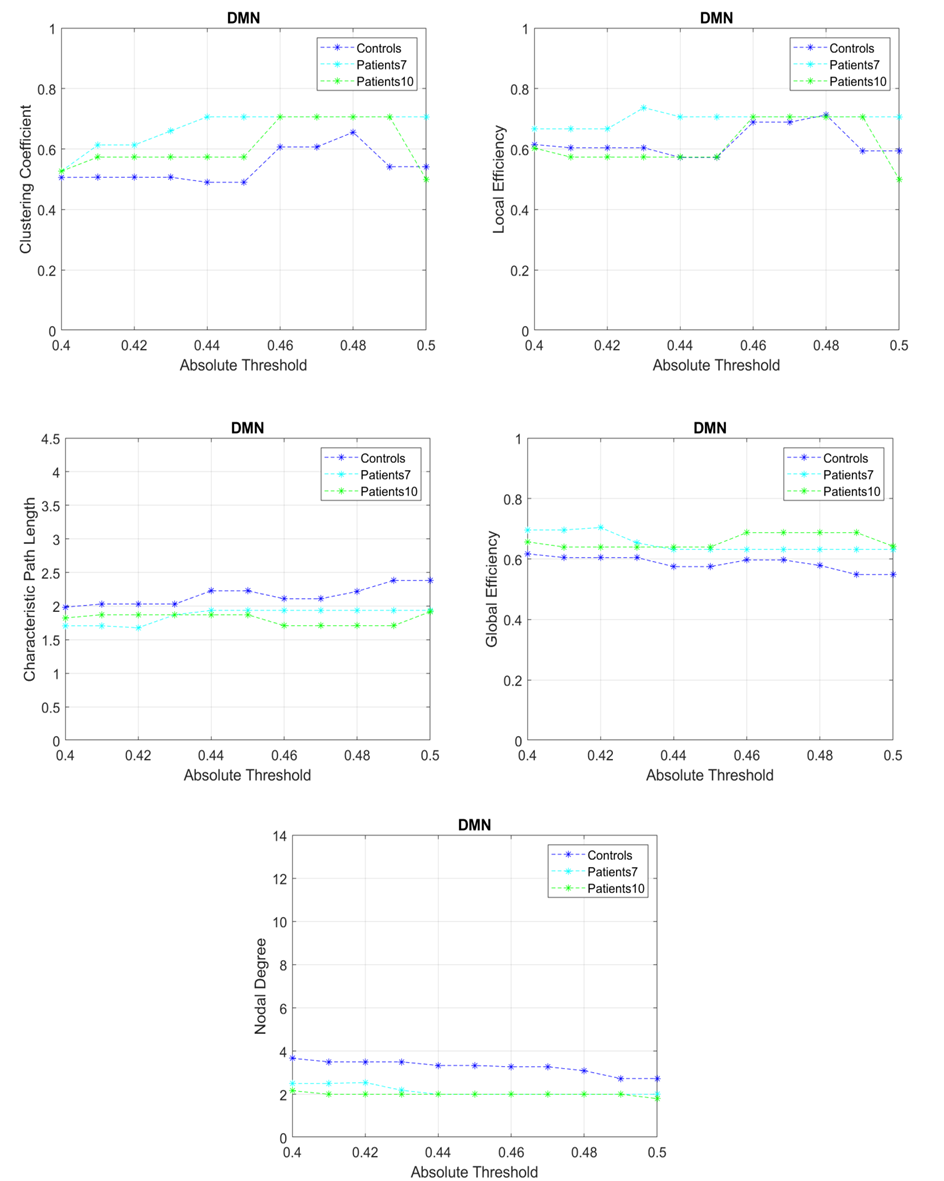

Supplement: S3 Fig — Sensitivity analysis showing changes in the graph measures extracted from the adjacent unweighted undirected matrices of the Default Mode Network (DMN), according to the absolute threshold applied to the pairwise correlation coefficients, within the range r = 0.4–0.5. (TIF) [file pone.0226816.s008.tif]

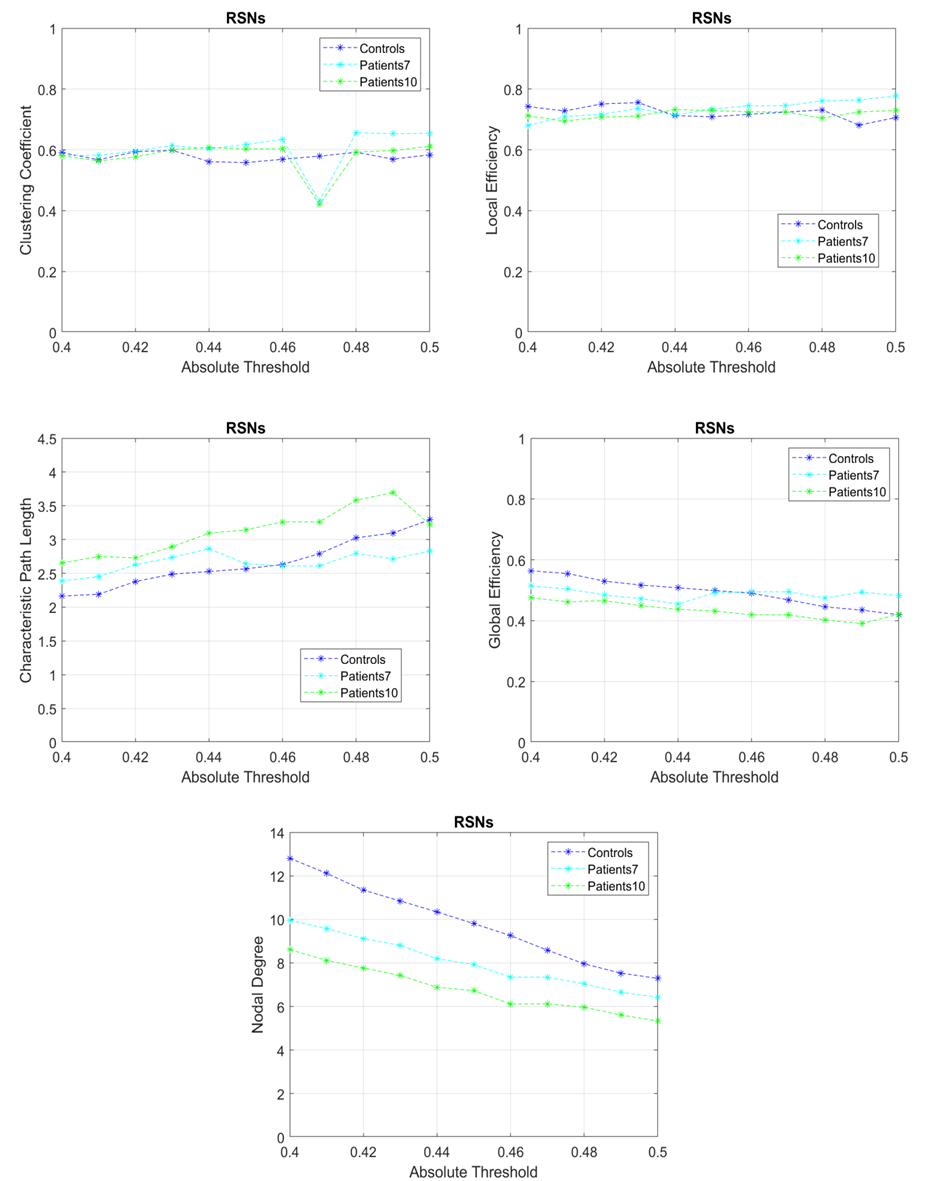

Supplement: S4 Fig — Sensitivity analysis showing changes in the graph measures extracted from the adjacent unweighted undirected matrices considering the inter-network functional connectivity, according to the absolute threshold applied to the pairwise correlation coefficients, within the range r = 0.4–0.5. (TIF) [file pone.0226816.s009.tif]

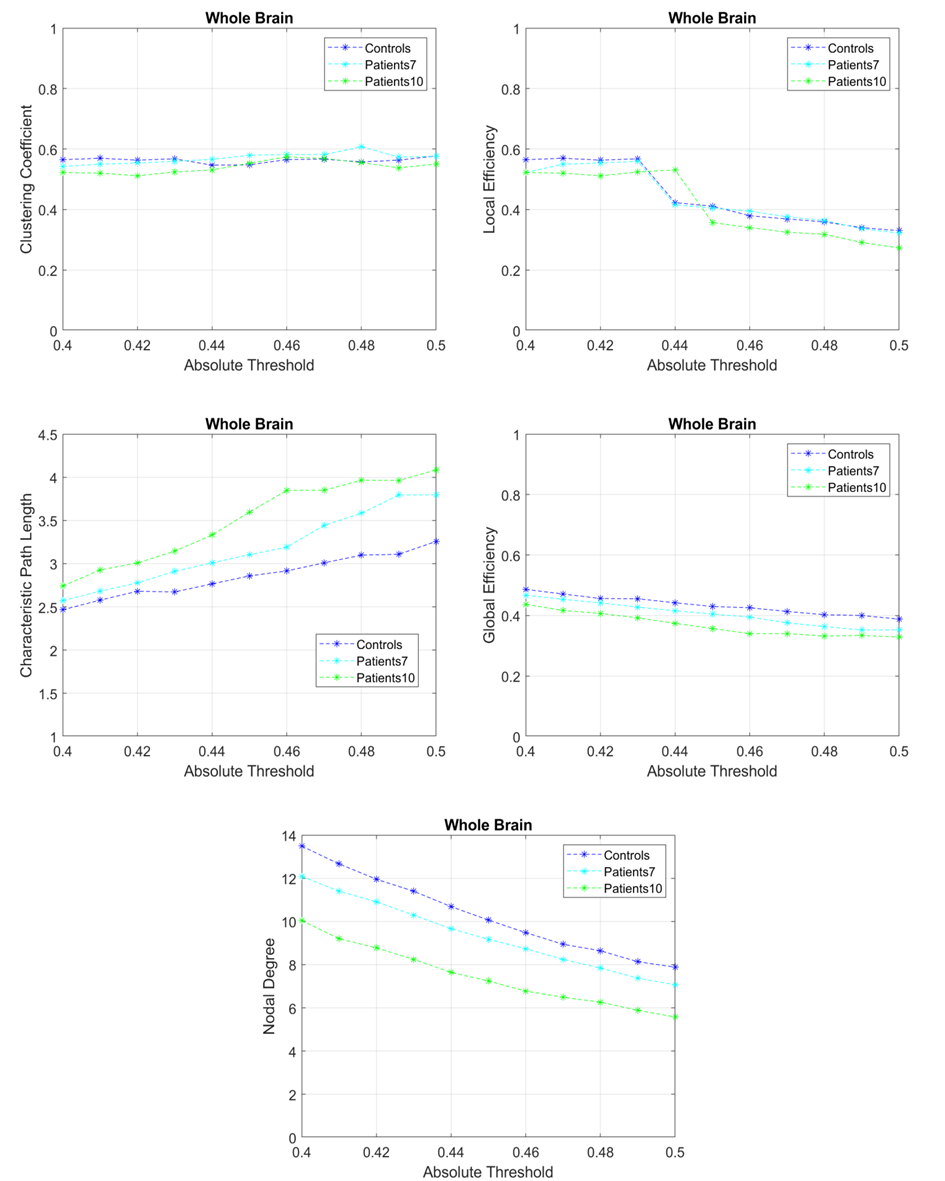

Supplement: S5 Fig — Sensitivity analysis showing changes in the graph measures extracted from the adjacent unweighted undirected matrices considering whole brain functional connectivity, according to the absolute threshold applied to the pairwise correlation coefficients, within the range r = 0.4–0.5. (TIF) [file pone.0226816.s010.tif]
